# Recent Therapeutic Improvements in Pediatric Oncology: One Fellow Generation (2020–2025)

**DOI:** 10.3390/cancers18213447

**Published:** 2026-10-25

**Authors:** Diannet Quintero-García, Aina Seguí, Andrea G. Palau, Alicia Pablos, Mariona Nadal, Ana I. Vega, Itziar Bueno, Miriam Dacil Cañada, Raquel P. Suarez, María Iriondo, Martín Cueto, Maite Gorostegui, Alicia Castañeda, María Muñoz, Ivan Prieto, Veronica Celis, Paula Mazorra, Juan P. Muñoz, Ainhoa Lasarte, Marina Caballero-Bellón, Cristina Larrosa, Jaume Mora

**Affiliations:** 1 Fellowship Program, Pediatric Cancer Center Barcelona (PCCB), Hospital Sant Joan de Déu, 08950 Esplugues de Llobregat, Barcelona, Spain; diannet.quintero@sjd.es (D.Q.-G.);; 2 Pediatric Cancer Center Barcelona (PCCB), Hospital Sant Joan de Déu, 08950 Esplugues de Llobregat, Barcelona, Spainmarina.caballero@sjd.es (M.C.-B.); 3 Pharmacy, Pediatric Cancer Center Barcelona (PCCB), Hospital Sant Joan de Déu, 08950 Esplugues de Llobregat, Barcelona, Spain

**Keywords:** new oncological agents, pediatric oncology, pediatric cancers, targeted agents, precision pediatric oncology

## Abstract

Recent therapeutic advances have significantly transformed the management of selected pediatric malignancies. We conducted a narrative review of recently approved therapies with demonstrated clinical impact in pediatric oncology. Evidence from pivotal clinical trials was integrated with real-world experience at the Pediatric Cancer Center Barcelona (PCCB). Fifty-three new oncological drugs with pediatric indication were available in 2025 that were not in routine clinical practice in 2020. We reviewed 18, those available at the PCCB, to demonstrate their meaningful clinical activity, the improved disease control and favorable toxicity profiles. Overall, 688 patients have benefited from these new agents. Real-world experience supports their feasibility and clinical benefit in many pediatric cancers. We realized that a significant number of novel therapies have been approved in the last 5 years and represent an important step toward precision oncology in pediatric cancer. Their integration into clinical practice is reshaping treatment paradigms and offering real opportunities for patients with otherwise limited options. Ongoing studies will define their long-term impact, optimal sequencing, and role within multimodal treatment strategies.

## Simple Summary

Pediatric cancers are rare diseases and development of specific new therapies has been historically very slow or absent. In this review, we describe recently approved (2020–2025) therapies that have been shown to impact outcomes of patients. Evidence from key clinical trials is integrated with real-world experience at the Pediatric Cancer Center Barcelona (PCCB). We reviewed 18 new drugs that became available at the PCCB in the last 5 years, to show their meaningful clinical activity, improved disease control and favorable toxicity profiles. We show how 688 patients have benefited from these new agents. In this review we realized that a significant number of novel therapies have been approved in the last 5 years and represent an important change from previous times and a significant step toward precision oncology in pediatric cancer.

## 1. Introduction

Pediatric cancers are a group of tumors characterized by rarity and a link to developmental biology. They usually present in the first two decades of life, although not exclusively. Cancers affecting this population (children, adolescents and young adults) are the leading cause of death within this age group in high-income countries (HICs), accounting in total for less than two percent of all cancers [1,2]. Most pediatric cancers are qualitatively different from those that affect adults, and compelling evidence on the fundamental differences between pediatric and adult cancer derives from the fact that the few cancer types that occur in both children and adults present marked differences in terms of molecular landscape and malignant potential depending on the age of the patient [3,4,5]. Hence, the types of cancer that affect the under-20 population are different to those that affect adults and, in stark contrast to them, are rare. The most common pediatric cancers include acute leukemias, brain tumors, lymphomas, bone and soft tissue sarcomas, and germ cell tumors. The typical “embryonal tumors” (leukemia, neuroblastoma, renal tumors, retinoblastoma) are confined largely to younger children, whereas cancers in adolescents include musculoskeletal tumors, lymphomas and melanoma [6]. An estimated 429,000 new cases of pediatric cancer are expected globally each year. The age-standardized reported incidence rates are 141 per million person-years for children and 185 per million person-years for adolescents and young adults [7]. Age-specific incidence rates vary by geography and ethnicity.

Pediatric oncology has benefitted greatly from a multidisciplinary approach to treatment, with resultant general improvement. In recent years, 5-year survival rates have risen to ~80% in HICs [8]. This progress reflects the optimized use of conventional therapies (e.g., cytotoxic drugs) through better risk stratification of patients. For example, based on molecular prognostic markers, treatments are intensified for patients identified as high-risk and deescalated for patients identified as low-risk to reduce the likelihood of immediate and long-term side effects. Expanding portfolios of new drugs that target the biological mechanisms driving the growth of pediatric cancers are starting to contribute to improved cure rates. However, until 2020 few children with cancer were treated with targeted agents as standard of care in the frontline/curative-intent setting. As of 2020, only patients with Philadelphia chromosome-positive acute lymphoblastic leukemia (Ph+ ALL) and chronic myelogenous leukemia (CML) were treated with ABL-class inhibitors; acute promyelocytic leukemia (APML) received ATRA; B-cell lymphomas were treated with anti-CD20 antibodies; high-risk neuroblastoma (HR-NB) was managed with anti-GD2 antibodies; and TRK fusion cancers could receive TRK inhibitors [9].

Genomic information is increasingly being used in the clinic to make treatment decisions for individual patients. Over the past decade, modern genomic technology has enabled accumulation of massive amounts of data on the molecular underpinnings of tumor development and growth. Efforts like The Cancer Genome Atlas (TCGA) research network, launched by the USA National Institute of Health (NIH) in 2009, aimed to explore the entire spectrum of genomic changes involved in human cancer. TCGA and other researchers, including many pediatric oncology focused teams, are charting genomic changes in all different cancer types. Their findings are made readily available to the worldwide research community, accelerating development of new targeted therapies. From January 2020 until December 2025, based on compelling evidence of benefit shown in clinical trials, the US Food and Drug Administration (FDA) approved 96 new anticancer drugs to treat patients with advanced and/or treatment-resistant cancer including breakthrough therapy designation, established in 2012, to speed development of new treatments that may lead to substantial improvement over existing therapies (Table 1). Of these 96 newly approved oncological drugs, 53 (55%) had pediatric approval. Most of the newly approved agents are targeted drugs that block the activity of specific proteins (i.e., kinases) that fuel cancer growth. Approvals include agents in other drug classes like radiopharmaceuticals and immunomodulatory agents.

The conventional model of drug development had been histology-based. Instead, tumor-agnostic drug development of a new drug (or combination) focuses on targeting a common genomic biomarker in multiple cancers, regardless of histology. The basket-like clinical trials with multiple cohorts allow clinicians to evaluate pan-cancer efficacy and toxicity. There are currently eight tumor-agnostic approvals granted by the FDA, including two immune checkpoint inhibitors (ICIs), and five targeted therapy agents. Pembrolizumab is an anti-programmed cell death protein-1 (PD-1) antibody that was the first FDA-approved tumor-agnostic treatment in 2017 [10,11]. In 2021, another anti-PD-1 antibody, dostarlimab, also received FDA accelerated approval for patients with mismatch repair-deficient (dMMR) recurrent or advanced solid tumors, becoming the second tissue-agnostic PD-1 inhibitor approved by the FDA [12,13]. Larotrectinib and entrectinib received tissue-agnostic FDA approval for the treatment of neurotrophic tyrosine receptor kinase (NTRK) fusion-positive solid tumors, with larotrectinib becoming the first therapy approved for a specific genomic alteration irrespective of tumor histology [14,15,16,17]. Later, selpercatinib was approved for Rearranged during Transfection (RET) fusion-positive solid tumors [18,19]. The FDA approved dabrafenib plus trametinib as the first tumor-agnostic combination therapy for patients 6 years or older with unresectable or metastatic solid tumors carrying a BRAF V600E mutation (reviewed in Section 2.2) [20]. The most recent FDA tumor-agnostic approval is fam-trastuzumab deruxtecan-nxki (T-Dxd), the first antibody–drug conjugate (ADC) approved for HER2-positive solid tumors [21,22].

This paradigm shift toward biomarker-driven treatment has led to the approval of several targeted agents with broad applicability across malignancies. In pediatric oncology, where actionable genomic alterations are often key drivers of disease, this approach is particularly relevant [23,24]. The identification of molecular targets has facilitated the use of selective inhibitors with improved efficacy and tolerability compared to conventional cytotoxic therapies.

To analyze how much pediatric cancer patients benefit from the newly available drugs, we put ourselves a question: how many new drugs have we incorporated into the armamentarium of our pediatric cancer referral center over the last 5 years, about one generation of pediatric oncology fellows? We analyzed the new drugs that became available for routine use in our center during the interval period of 2020 to 2025, to discover how many patients benefitted from these new opportunities. Despite the many difficulties in the field outlined in several good reviews [9], we were surprised to find that 53 new drugs with pediatric indication were approved in 2025 that were not in routine clinical practice in 2020 (Table 1). One generation of fellows in pediatric oncology had been sufficient to generate real novel opportunities for many pediatric cancer patients, strong data to support an optimistic view for the new generations. The analysis of the new drugs made available to the pediatric cancer armamentarium, and the way they reached the market, may support pragmatic designs for the new developments currently in the pipeline of both pharmaceutical companies and academic teams. 

In this review, we focus on 18 agents approved since 2020 that are now routinely used at the PCCB, having demonstrated clinically relevant impact on disease courses of pediatric cancers. By integrating evidence from clinical trials with real-world experience, we aim to provide a practical overview of how these therapies are reshaping treatment paradigms.

A summary of the 18 new drugs reviewed in this article is provided in Table 2. This list of new agents was selected purely based upon drug availability and includes 13 targeted drugs that block the activity of specific proteins (mostly kinases); one radiopharmaceutical; and four immunotherapeutic agents.

## 2. Targeted Drugs

### 2.1. Nirogacestat for Desmoid Tumors

Desmoid tumors (DTs), also known as aggressive fibromatosis, are rare, locally aggressive soft tissue neoplasms that occur in both children and adults, with a peak incidence in young adults. Increased activity of β-catenin is the hallmark and driving force of DTs, which are monoclonal fibroblastic neoplasms characterized by locally infiltrative growth and lack of metastatic potential [25]. DTs arise in two distinct clinical settings: sporadically or in association with familial adenomatous polyposis (FAP). FAP is an autosomal dominant hereditary cancer predisposition syndrome caused by germline pathogenic variants in the adenomatous polyposis coli (APC) gene. In contrast, approximately 85–90% of sporadic DTs harbor somatic mutations in the CTNNB1 gene, which encodes β-catenin. Although the underlying genetic alterations differ, both mechanisms result in aberrant activation of the Wnt/β-catenin signaling pathway.

For most patients, DTs are chronic and symptomatic diseases, which affect health-related quality of life. Compression of vital structures can result in pain, loss of function, and nerve damage, significantly impacting patients’ quality of life. The management of DTs remains challenging, as treatment decisions are individualized according to patient age, tumor location, symptoms, and the rate of disease progression. Surgery, once the cornerstone of treatment, is now recommended less frequently because of its associated morbidity and high post-surgical recurrence rates, particularly in infiltrative or anatomically challenging tumors. These challenges have fueled the development of novel systemic therapies, such as molecularly targeted agents, which have transformed the management of DTs and are redefining the treatment paradigm for patients with progressive disease [26,27]. 

Nirogacestat (PF-03084014, IND# 146375, SpringWorks Therapeutics, Inc. Ogsiveo^®^) is an oral, selective, reversible γ-secretase inhibitor approved by the FDA in November 2023 for adults with progressing DTs requiring systemic therapy, representing the first FDA-approved drug specifically for DTs [28,29]. By inhibiting the γ-secretase, nirogacestat prevents the release of the Notch intracellular domain, thereby suppressing Notch signaling and downstream transcriptional programs involved in tumor cell proliferation. Given the extensive crosstalk between the Notch and Wnt/β-catenin pathways, γ-secretase inhibition may also influence Wnt signaling, providing a biological rationale for its activity in DTs driven by CTNNB1 or APC mutations [30].

The pivotal DeFi trial was an international, multicenter, randomized, double-blind, placebo-controlled phase III study that enrolled 142 adults with progressing DTs requiring systemic therapy [28]. Patients were randomized 1:1 to receive nirogacestat 150 mg or placebo orally twice daily in continuous 28-day cycles. Nirogacestat demonstrated significant progression-free survival (PFS) benefit over placebo, with an estimated 2-year PFS rate of 76% versus 44%. The objective response rate (ORR) was 41% versus 8% with placebo, including complete responses (CR) in 7% of nirogacestat-treated patients. Long-term follow-up, with median exposure of 33.6 months, demonstrated sustained efficacy, with ORRs increasing to 45.7% after up to 4 years of treatment and 72% of patients achieving a clinically meaningful reduction in pain compared with 29% in the placebo group [31]. Further tumor size reduction occurred in most patients, and patient-reported outcome benefits were sustained. The safety profile showed that 95% of adverse events (AEs) were grades 1–2. The most frequent ones were diarrhea, nausea, fatigue, hypophosphatemia, and maculopapular rash. Among women of childbearing potential, 75% experienced ovarian dysfunction, which resolved in 74% of cases [28]. Long-term safety data demonstrated that AEs decreased in incidence and severity over time.

Following its FDA approval, nirogacestat was incorporated into the NCCN Clinical Practice Guidelines for Soft Tissue Sarcoma as a Category 1 preferred regimen for DTs [32]. Given its superiority in efficacy and favorable safety profile demonstrated in the DeFi trial, nirogacestat may become first-line treatment when medical therapy is indicated. Compared to tyrosine kinase inhibitors (TKIs) and conventional chemotherapy, nirogacestat offers superior efficacy with better tolerability. The oral administration and absence of significant cumulative toxicity allow patients to maintain daily routines and reduce healthcare burden.

The safety and efficacy of nirogacestat in pediatric patients has not been fully established. The FDA label warns against its use in children from birth to less than 2 years due to potential safety concerns related to structural and functional growth. Limited evidence from compassionate use reports four pediatric/young adult patients who received Nirogacestat with durable clinical benefit and no grade 3–4 AEs [33].

The approval of nirogacestat has fundamentally changed the therapeutic landscape of DTs, establishing γ-secretase inhibition as a new standard of care and opening new avenues for targeted treatment [34]. The Children’s Oncology Group (COG) is currently formally testing Nirogacestat in the population aged less than 18 years, in clinical trial ARST1921.

#### PCCB Experience

**Figure 1:**
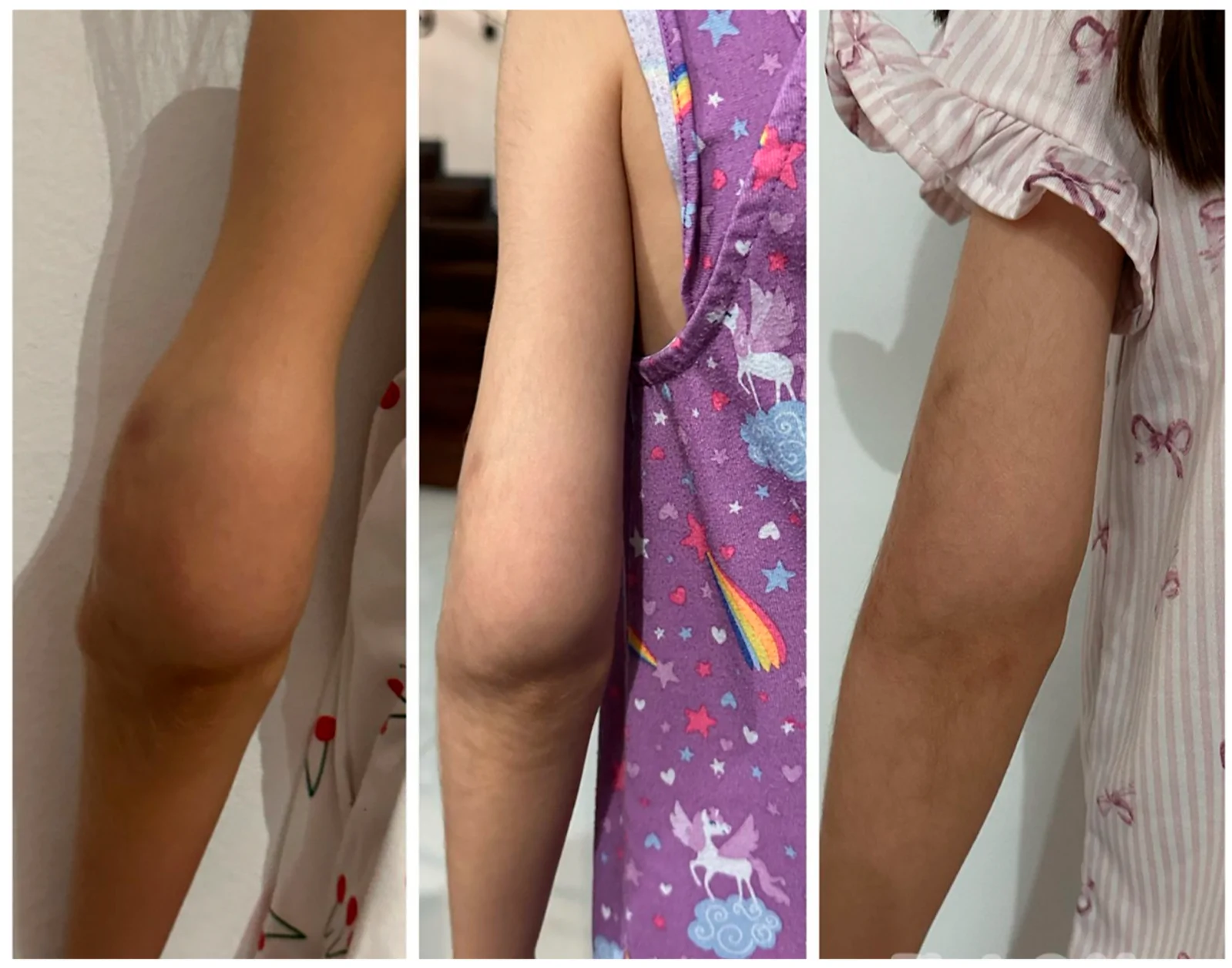
Eight-year-old female patient with progressing DT in the left arm. Treatment with Nirogacestat resulted in clear clinical responses. Images from 1 and 2 months after single-agent Nirogacestat.

Three patients, ages 8, 13, and 17, with progressing DTs are currently receiving nirogacestat therapy under compassionate use approved by the Spanish National Health Authorities (AEMPS). Favorable clinical responses with good tolerability have been documented in all of them, supporting the potential role of this agent in selected pediatric cases. Figure 1 shows the clinical response of an 8-year-old child with progressing DT in the left arm after 2 months of treatment with nirogacestat. The only patient that has experienced AEs is the 17-year-old, with grade 1 nausea and grade 3 ALT and AST increases, both resolved after one level dose decrease.

### 2.2. MAPK Pathway Inhibitors

#### 2.2.1. Dabrafenib and Trametinib for Low-Grade Gliomas

Pediatric low-grade gliomas (LGGs) and glioneuronal tumors, defined as World Health Organization (WHO) grade I or II lesions of the central nervous system (CNS), are an extremely diverse group of tumors. Together they comprise approximately one-third of all brain tumor diagnoses in children, making them the most common CNS neoplasia in pediatrics. Most pediatric LGGs are caused by one of a variety of alterations in the signaling pathway of mitogen-activated protein kinase (MAPK), including BRAF mutation or fusion, fibroblast growth factor receptor 1 (FGFR1) mutation or structural rearrangement, NF1 mutation, NTRK-family fusions, and other rarer events. Genetic alterations that result in constitutive activation of the BRAF kinase, including a nucleotide transversion resulting in the substitution of valine (V; most commonly with glutamic acid (E) at position 600 (i.e., V600E point mutation) or a tandem duplication resulting in the fusion of KIAA1549 and BRAF (i.e., BRAF:KIAA1549)), are the most common aberrations implicated in the development of pediatric LGGs [35,36,37].

The development of drugs specifically targeting several of these alterations (e.g., BRAF V600E, FGFR1, NTRK) or the downstream mediators of the pathway (e.g., MEK) prompted clinical trials testing specific MAPK inhibitors in this disease [35,36]. In 2023, the FDA approved the combination of Dabrafenib and Trametinib for pediatric patients aged 1 year and older with LGGs harboring a BRAF V600E mutation that requires systemic therapy [38,39].

Dabrafenib (Novartis; Tafinlar^R^) is a potent and selective inhibitor of the BRAF kinase, which suppresses ERK signaling downstream of BRAF in the MAPK pathway. Dabrafenib showed anti-proliferative activity against multiple tumor cell lines harboring the BRAF V600E mutation [40]. 

Trametinib (Novartis, Mekinist^R^) is a reversible, highly selective allosteric inhibitor of MEK1/MEK2, inhibiting both MEK activation and kinase activity, thereby suppressing downstream ERK signaling within the MAPK pathway [41].

Historical management for patients whose LGG is not amenable to definitive surgery or whose tumors have recurred or progressed included chemotherapy and radiotherapy, which provided 3-year EFS rates up to approximately 70% but were associated with significant morbidities. Standard therapy included combination of carboplatin and vincristine, or weekly vinblastine [42,43]. In 2023, a phase II randomized study reported the combination of dabrafenib and trametinib with significantly higher ORR, more prolonged EFS, and better safety profile compared to standard chemotherapy in first-line therapy for pediatric patients with BRAF V600E-mutant LGGs [38]. Many patients experienced marked reductions in tumor volume, leading to improved symptom control and quality of life. The profile of AEs included skin toxicity and gastrointestinal symptoms, although generally grades 1–2. 

##### PCCB Experience

At the PCCB we integrated the combination of dabrafenib and trametinib into our standard clinical practice in 2020. For children with confirmed BRAF V600E LGG who show clinical signs of progressive disease or have significant residual tumors, particularly those with symptoms or those at high risk of losing vision or neurological functions, we start with dabrafenib as monotherapy and keep the combination for cases that progress on it. We have treated 22 patients with BRAF-mutated LGG using targeted therapy. Our experience shows a significant response rate, with 77% (17/22) disease control rate (DCR; including stable disease) for dabrafenib monotherapy, with manageable side effects that do not significantly impact the overall treatment course of most patients. In three cases, the addition of trametinib was required due to disease progression, subsequently achieving disease control. In two additional cases, surgical intervention was performed. 

**Figure 2:**
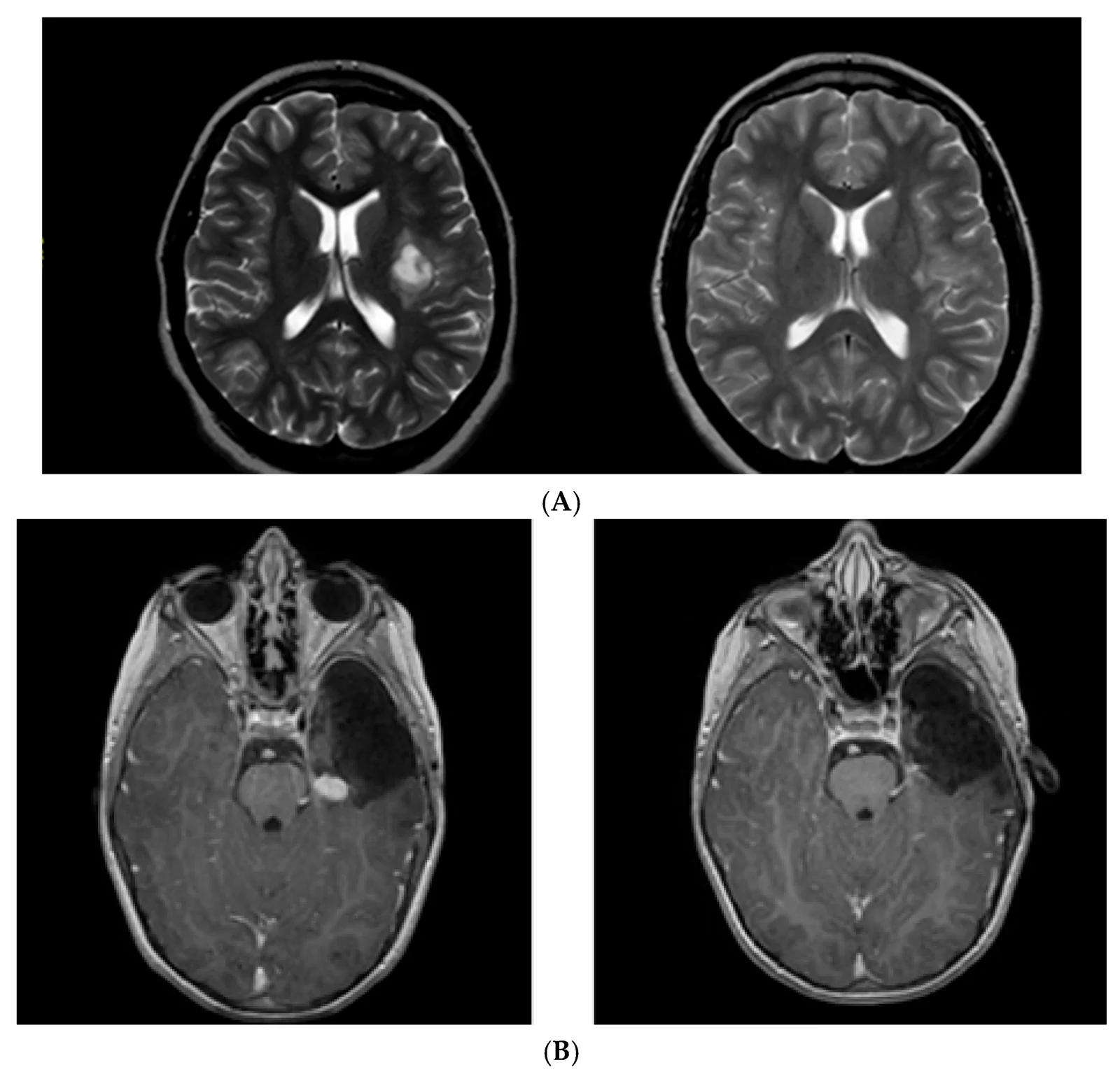
(**A**) MRI of six-month-old patient with BRAF V600E-mutant, CDKN2A-deletion pilocytic xanthoastrocytoma (PXA) showing complete response after three months of treatment with single-agent dabrafenib. (**B**) MRI of 15-year-old patient with BRAF V600E-mutant, CDKN2A-deletion, subcortical, left insula Ganglioglioma showing partial response after six months of therapy with single-agent dabrafenib.

Figure 2 shows two examples of objective responses with single-agent dabrafenib: the MRIs of a 6-month-old patient with BRAF V600E-mutant, CDKN2A-deletion pilocytic xanthoastrocytoma showing complete response after three months of single-agent dabrafenib, and the MRI of a 15-year-old patient with BRAF V600E-mutant, CDKN2A-deletion, subcortical, left insula Ganglioglioma with partial response after six months of treatment.

Our administration regimen is oral Dabrafenib 5.25 mg/kg/day once daily and oral Trametinib 0.032 mg/kg/day every 12 h. After 2 years of treatment, we start gradual reduction of therapy or treatment discontinuation.

#### 2.2.2. Tovorafenib for LGGs

Tovorafenib (Day One Biopharmaceuticals; Ojemda^R^) is an orally administered, CNS-penetrant, selective pan-RAF kinase inhibitor authorized for pediatric patients aged ≥6 months with relapsed or refractory (R/R) LGG harboring BRAF fusion, BRAF rearrangement, or BRAF V600 mutation [44].

**Figure 3:**
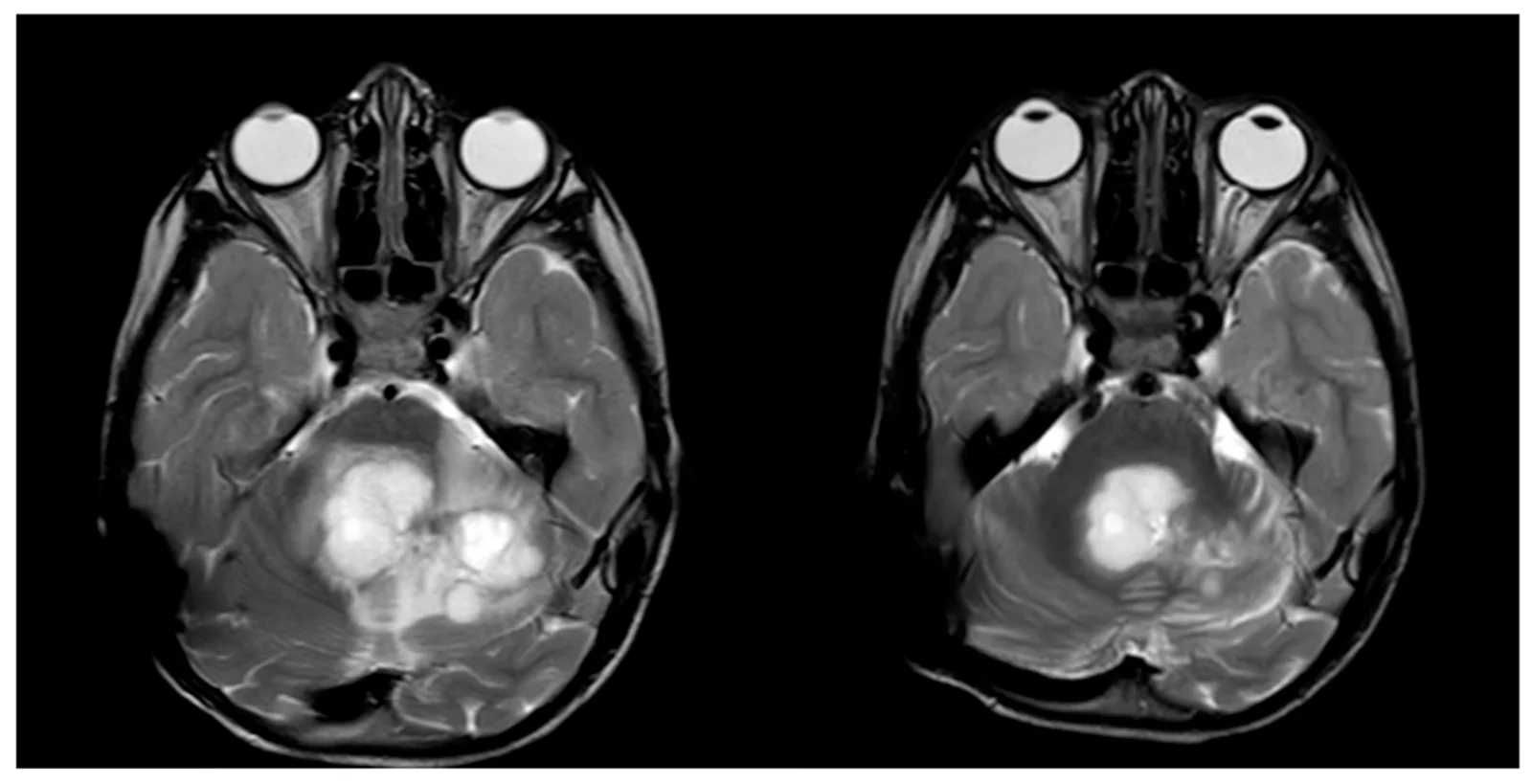
MRI of a 5-year-old patient with LGG after 12 weeks of treatment showing a 31% reduction in tumor size, corresponding to a minor response according to the RANO-LGG criteria.

Tovorafenib is a type 2 RAF inhibitor, binding RAF kinases and suppressing RAS–RAF–MEK–ERK cascade signaling [45,46]. The agent demonstrated activity against BRAF V600 mutation and BRAF fusion/rearrangement alterations, without triggering the paradoxical MAPK pathway activation associated with certain type 1 RAF inhibitors. This distinction is clinically relevant, as the first approved targeted therapy for pediatric LGG with BRAF V600 mutations—dabrafenib in combination with trametinib (see Section 2.2.1)—was indicated for patients older than one year and has not demonstrated clinical efficacy in tumors driven by BRAF fusions or rearrangements. Consequently, tovorafenib has expanded the population of patients eligible for targeted therapy among children with RAF-altered LGG (Figure 3).

In April 2024, Tovorafenib received accelerated FDA approval, based on the multi-center, open label, single-arm phase II FIREFLY-1 trial (PNOC026; NCT04775485) in children and young adults with R/R BRAF-altered pediatric LGG [47]. The approval was supported by an independently assessed objective response rate of 51% according to Response Assessment in Neuro-Oncology for LGG (RANO-LGG) criteria. The primary publication of FIREFLY-1 also reported an ORR of 67% using RANO for high-grade glioma (RANO-HGG criteria), with a median duration of response of 16.6 months and median time to response of 3 months. These responses were both clinically meaningful and durable across patients with BRAF fusions and V600 mutations [48].

Tovorafenib represented another step forward in the pediatric LGG therapeutic landscape, addressing a major unmet need for children whose tumors are unresectable or recurrent after standard therapy. The promising efficacy, together with the favorable safety and tolerability profile of tovorafenib, led to the initiation of the ongoing phase III LOGGIC/FIREFLY-2 trial. This study compares tovorafenib with the investigator’s choice of standard chemotherapy, including COG vincristine plus carboplatin (60 weeks), SIOPE-LGG vincristine plus carboplatin (81 weeks), or vinblastine monotherapy (70 weeks) [49]. 

A range of AEs were associated with Tovorafenib in the FIREFLY-1 trial, including dermatologic toxicities and hair-pigmentation changes, fatigue and epistaxis. Less frequent but clinically relevant AEs included reduced growth velocity and tumor hemorrhage. Grade ≥3 laboratory abnormalities included anemia, increased creatine phosphokinase and transaminase elevation [48].

##### PCCB Experience

The LOGGIC/FIREFLY-2 trial ended patient recruitment in April 2026, including seven patients from our institution. Currently, five remain on active treatment and two are in follow-up. In contrast to most standard chemotherapy regimens that require intravenous administration, patients receiving tovorafenib take an oral agent once weekly at home. The medication is available as tablets and oral suspension, which can be administered through a nasogastric or gastric tube, thereby facilitating the continuation of routine daily activities, including school attendance. 

Tovorafenib is currently approved under the FDA Accelerated Approval Program for pediatric patients with R/R LGG harboring an activating BRAF alteration. It may become a promising first-line therapy. However, results from ongoing randomized clinical trials comparing tovorafenib with conventional chemotherapy are needed to confirm its role in the frontline setting. Clinical experience with tovorafenib in adults remains limited because BRAF-altered low-grade gliomas occur predominantly in children, whereas adult tumors are more frequently driven by IDH1/2 mutations.

#### 2.2.3. Cobimetinib for Histiocytic Disorders

Histiocytic neoplasms such as Langerhans cell histiocytosis (LCH) and Erdheim–Chester disease (ECD) are recognized monoclonal disorders driven by persistent activation of the MAPK pathway, making histiocytoses targetable neoplastic diseases. This unified etiological understanding of histiocytoses began with the discovery of somatic BRAFV600E in ∼50% of patients with LCH and ECD. Subsequent genomic studies identified a broad spectrum of activating alterations in patients lacking BRAFV600E, including mutations in MAP2K1 (encoding MEK1), MAP2K2 (encoding MEK2), ARAF, CSF1R, N/KRAS, or PIK3CA, as well as activating BRAF, ALK, RET, and NTRK1 fusions and in-frame BRAF deletions [50,51,52].

The discovery of BRAFV600 mutations in patients with histiocytoses led to the development of clinical trials targeting BRAF in adults with histiocytic neoplasms. In the pivotal phase II VE-BASKET trial, vemurafenib achieved an ORR of 100% in patients with *BRAF* V600-mutant ECD, leading to its FDA approval for *BRAF* V600-mutant ECD in 2017 [53,54]. Given the excellent efficacy of vemurafenib for BRAFV600-mutant histiocytoses and the near-universal presence of mutations activating ERK in patients with histiocytoses, MEK1/2 inhibitor therapy in BRAFV600-wild-type histiocytoses was investigated. In the pivotal open-label phase II trial, single-agent MEK1/2 inhibitor, cobimetinib, achieved an ORR of 83% by PET-based response criteria in adults with histiocytic neoplasms. Responses were observed across the spectrum of histiocytosis subtypes and in both *BRAF* V600-wild-type and *BRAF* V600-mutant disease, with a median response duration exceeding 30 months. These findings established MAPK/ERK signaling as a shared oncogenic dependency and actionable therapeutic target across histiocytic neoplasms, leading to FDA approval of cobimetinib for adults with histiocytic neoplasms in 2022 [55].

The efficacy of MEK inhibitors has been consistently reported in other prospective and retrospective studies, as well as in multiple case reports of LCH, ECD, Rosai–Dorfman disease, and related histiocytic neoplasms [56,57]. Across these reports, MEK inhibitors have been associated with objective responses, leading to improvements in organ involvement—including pulmonary, hepatic, skeletal, CNS, and hematopoietic systems—as well as disease-related clinical manifestations. Importantly, clinical experience suggests that disease recurrence may occur following treatment discontinuation, highlighting the potential need for continuous therapy to maintain response. These observations indicate that the optimal duration of MEK inhibitor therapy remains undefined, and long-term follow-up is necessary to assess both sustained efficacy and potential late effects.

Cobimetinib exhibits a well-established safety profile, with most common AEs comprising diarrhea, rash, photosensitivity, nausea, vomiting, fatigue, and pyrexia. Laboratory abnormalities reported include elevated liver enzymes, increased creatine phosphokinase, lymphopenia, and electrolyte disturbances. Serious toxicities described in product labeling include decreased left ventricular ejection fraction and serous retinal disorders, which should be monitored with appropriate cardiac and ophthalmologic assessments during treatment. Most AEs are manageable with dose modification or supportive care.

##### PCCB Experience

We recently treated one infant with severe pulmonary LCH refractory to three prior lines of therapy including MEK inhibitor trametinib. Repeated molecular profiling by next-generation sequencing (NGS) identified MAP2K1 p.P105_A106del mutation. Preclinical data suggest that this mutation confers reduced sensitivity to trametinib compared to cobimetinib [58]. Also, published case reports in histiocytic disorders have described significant clinical benefits with cobimetinib in similar settings [59,60,61,62]. Based on these findings, we initiated treatment with cobimetinib, leading to a marked clinical improvement allowing the discontinuation of advanced respiratory support measures. This experience underscores the importance of mutation-specific MEK inhibitor selection and the need for repeated molecular testing in refractory histiocytosis.

#### 2.2.4. MEK Inhibitors for NF1-Associated Plexiform Neurofibromas: Selumetinib and Mirdametinib

Neurofibromatosis type 1 (NF1) is caused by loss-of-function mutations in the NF1 gene, which encodes neurofibromin, a negative regulator of RAS. Loss of neurofibromin results in sustained activation of the RAS–RAF–MEK–ERK cascade, a central regulator of cell growth, proliferation, and survival. Within this module, the RAF isoforms cooperate with downstream MEK 1/2 and ERK1/2 kinases to regulate the physiological processes. In NF1, this dysregulation promotes Schwann cell proliferation and drives the development of plexiform neurofibromas (PN), histologically benign peripheral-nerve sheath tumors present in up to 50% of NF1-affected individuals, which can cause substantial morbidity given their predominant growth during early childhood [63,64].

Within the MAPK cascade, MEK1/2 act as the sole activators of ERK1/2; therefore, pharmacologically blocking MEK1/2 represents an efficient strategy to interrupt downstream signaling regardless of the specific upstream signal. However, the clinical efficacy of MEK inhibitors is highly genotype-dependent. Responses are greatest in *BRAF* V600-mutant tumors, in which MAPK signaling is constitutively activated without significant feedback reactivation. In contrast, responses in *NF1*-deficient, RAS-mutant, or RTK-driven tumors, such as *NF1*-associated PN, are generally less pronounced and more variable because of compensatory pathway reactivation through feedback mechanisms [65].

To date, the FDA has approved five MEK inhibitors: Trametinib (discussed in Section 2.2.1), Cobimetinib (discussed in Section 2.2.3), Binimetinib, Selumetinib [66] and Mirdametinib [67]. Selumetinib (AZD6244, Koselugo^R^) and Mirdametinib (Gomekl^R^/Ezmekly^R^) are both orally bioavailable, selective MEK1/2 inhibitors developed to interrupt the MAPK pathway in NF1-PN [68,69,70]. However, these agents differ in certain pharmacological properties. Although both Selumetinib and Mirdametinib are selective, non-ATP-competitive MEK1/2 inhibitors, Mirdametinib has demonstrated CNS penetration. In addition, preclinical studies in melanoma models have shown that Mirdametinib suppresses tumor neovascularization by a dual mechanism: ERK inhibition downregulates VEGF expression, thereby impairing endothelial proliferation and migration, while disruption of hypoxia-inducible factor (HIF) 1α stabilization blocks transcriptional activation of VEGF and PDGF, further limiting tumor neovascularization and modulating the tumor microenvironment [71].

##### Selumetinib (Astra Zeneca; Koselugo^R^)

The pivotal phase II SPRINT trial enrolled 50 children with NF1 and symptomatic, inoperable PNs treated with Selumetinib 25 mg/m^2^ twice daily, demonstrating tumor shrinkage in 68% of patients, meaningful improvements in pain, function, and quality of life, and a 3-year PFS of 84% versus 15% in the natural history cohort [68]. Long-term follow-up confirmed durable tumor shrinkage and sustained pain improvement over five additional years with no new safety signals, establishing the safety and tolerability of long-term treatment [72]. The phase I/II SPRINKLE trial subsequently validated a granule formulation with comparable pharmacokinetics, a manageable safety profile, and high palatability in children aged 1 to under 7 years unable to swallow capsules [73]. Finally, the phase III KOMET trial, the first randomized, placebo-controlled study in adults with FN1-associated PN, confirmed the efficacy of Selumetinib in adults, completing the clinical evidence supporting its use across the lifespan [74].

These data supported a series of landmark regulatory approvals, beginning with FDA approval in April 2020 as the first approved therapy for NF1-associated PN, followed by EMA approval in June 2021 for patients aged ≥3 years and subsequent label expansions to include children aged ≥1 year by both the FDA (September 2025) and EMA (January 2026) [66,75,76].

PCCB Experience

At the PCCB, 130 patients with NF1 have been treated with MEK inhibitors, including 90 of them who received Selumetinib as the primary indication for inoperable PN. Our clinical experience has expanded steadily, with more than 50 patients initiating selumetinib therapy over the past three years. Overall, 88% of treated patients demonstrated clinical benefit, including tumor shrinkage, pain relief, and improvement in functional impairment remaining on treatment. Selumetinib is routinely administered at a dose of 25 mg/m^2^ twice daily. Patients are monitored for AEs, most commonly dermatological and gastrointestinal toxicities, together with serial creatine kinase (CK) measurements, cardiac function assessments, and ophthalmological evaluations. Treatment discontinuation occurred in 12% of patients, equally attributable to treatment-related toxicity, lack of efficacy, and loss to follow-up (one third each).

**Figure 4:**
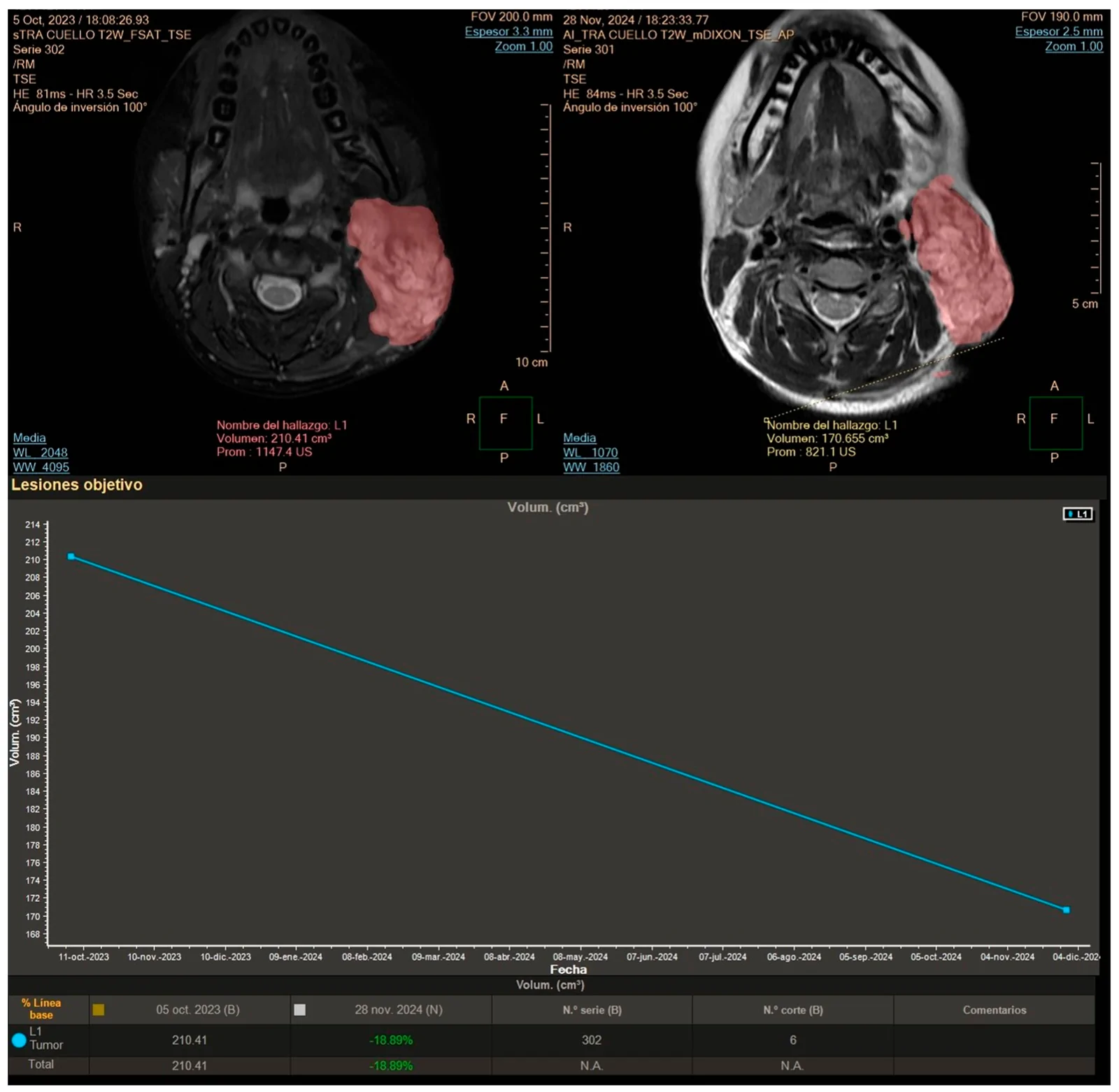
Shows MRI images and volumetric analysis of a cervical PN from an NF1 patient after one year of treatment with Selumetinib.

Figure 4 shows the MRI images and volumetric analysis of a cervical PN after one year of treatment with Selumetinib. Clinical improvement was observed, including pain reduction, with mild cutaneous toxicity.

##### Mirdametinib (SpringWorks Therapeutics; Gomekly^R^/Ezmekly^R^)

Mirdametinib was approved by the FDA in February 2025 for adult and pediatric patients aged ≥2 years with symptomatic, unresectable NF1-associated PN [67,77]. Approval was based on the pivotal multicenter, open-label, phase IIb ReNeu trial (NCT03962543), which enrolled both adult and pediatric patients (median pediatric age 10 years, range 2–17) with symptomatic NF1-PN causing significant morbidity [70]. The primary endpoint, ORR, defined as ≥20% reduction in target PN volume from baseline, was achieved in 52% of children and 41% of adults, with durable responses (≥12 months). Among responders, maximum tumor volume reduction exceeded 50% from baseline in 52% of children and 62% of adults. All pediatric patients (*n* = 56) reported at least one AE, mostly grade 1–2. The most frequent (≥20%) were acneiform dermatitis, diarrhea, paronychia, nausea, decreased ejection fraction, and increased blood CK.

Mirdametinib is currently being evaluated as monotherapy in a phase 1/2 trial in pediatric and adolescent patients with R/R LGGs (NCT04923126).

PCCB Experience

In our clinical practice, six patients have been treated with Mirdametinib. Four had NF1, whereas two patients without NF1 received the drug through an expanded access program for R/R KIAA1549-BRAF fusion-positive LGG. Among the four NF1-positive patients, indications comprised unresectable, painful PN (*n* = 3) and hypothalamic–chiasmatic glioma (*n* = 1). Mirdametinib was well tolerated, with AEs predominantly consisting of grade 1–2 dermatologic and gastrointestinal toxicities, and no grade ≥3 treatment-related AEs were observed.

### 2.3. Belzutifan for VHL-Related Tumors

Germline loss-of-function mutations of the von Hippel–Lindau (VHL) tumor suppressor gene cause von Hippel–Lindau disease, a hereditary cancer predisposition syndrome characterized by an increased risk of tumor development following somatic inactivation of the remaining wild-type VHL allele, consistent with Knudson’s two-hit hypothesis. The hallmark neoplasms of VHL disease include retinal, cerebellar, and spinal hemangioblastomas, clear cell renal cell carcinomas, and paragangliomas/pheochromocytomas [78,79]. 

The VHL encoded protein (pVHL) serves as the substrate recognition component of a multi-subunit ubiquitin ligase. The substrate most clearly linked to pVHL’s tumor suppressor activity is the HIF transcription factor. In addition, pVHL controls the activity of AKT. HIF is a heterodimeric transcription factor composed of an oxygen-sensitive labile α subunit (HIFα) and a constitutively expressed β subunit (HIFβ). Under normoxic conditions, pVHL recognizes hydroxylated HIFα, targeting it for ubiquitination and subsequent proteasomal degradation. In contrast, under hypoxic conditions or in the absence of functional pVHL, HIFα escapes degradation, accumulates within the cell, dimerizes with HIFβ, and activates the transcription of genes containing hypoxia response elements (HREs). Aberrant activation of the HIF pathway promotes angiogenesis, cell proliferation, metabolic reprogramming, and survival, thereby driving tumorigenesis. Consequently, individuals with VHL disease are predisposed to the lifelong development of multiple benign and malignant neoplasms [80,81]. 

Belzutifan (MSD; Welireg^R^) is a small molecule that selectively inhibits HIF-2α. It is the first HIF-2α inhibitor approved for clinical use in VHL disease-associated tumors [82]. It was approved by the FDA in 2021 for adults with VHL disease requiring therapy for associated renal cell carcinoma, CNS hemangioblastomas, or pancreatic neuroendocrine tumors (pNETs) not requiring immediate surgery [83]. In 2026, the indication was expanded to include patients aged ≥12 years with certain VHL-associated tumors, including pNETs. Pediatric use is supported primarily by the well-established molecular pathogenesis of VHL disease, together with pharmacokinetic and safety data extrapolated from adult studies, as pediatric-specific efficacy data remain limited.

The pivotal phase 2 LITESPARK trial enrolled patients with VHL disease-associated tumors who had not received prior systemic anticancer therapy. Belzutifan demonstrated robust antitumor activity, with an ORR of 91% in measurable pNETs, alongside substantial durable responses in CNS hemangioblastomas and clear cell renal cell carcinomas (ccRCC). The safety profile was manageable, with anemia and hypoxia as the most common AEs. Long-term follow-up confirmed sustained tumor control across VHL-associated neoplasms, supporting belzutifan as an effective strategy to delay or reduce the need for surgical intervention in adults [84,85,86,87].

Belzutifan has transformed the therapeutic landscape of VHL disease by providing the first effective systemic treatment option across multiple VHL-associated neoplasms. It has demonstrated durable antitumor activity while reducing the need for repeated surgical interventions. In pediatric patients, although clinical experience remains limited, belzutifan also seems to expand treatment options beyond surveillance and surgery, with a manageable toxicity profile.

#### PCCB Experience

At our institution, we have treated two adolescent patients (both aged ≥12 years) with progressive VHL-associated neoplasms: one with cerebellar hemangioblastoma and the other with ccRCC, pNET and leptomeningeal hemangioblastomas. Both patients have shown objective responses with a manageable safety profile, predominantly characterized by anemia and hypoxemia. At the time of writing, treatment durations are 10 and 9 months, respectively, with sustained clinical benefit, including reduced symptom burden, avoidance of additional surgical interventions and preservation of organ function.

### 2.4. Alpelisib for PIK3CA-Related Tumors

Activating mutations in PIK3CA lead to constitutive activation of the PI3K/AKT/mTOR signaling pathway. PIK3CA, which encodes the p110α catalytic subunit of phosphatidylinositol 3-kinase (PI3K), is among the most frequently mutated oncogenes across all human cancers (particularly H1047R, E542K, and E545K hotspot mutations). Aberrant activation of this pathway promotes tumorigenesis, treatment resistance, and enhanced cellular motility, contributing to an adverse clinical outcome in multiple malignancies [88]. In pediatric oncology, PI3K dysregulation has been documented across diverse tumor types including CNS tumors (medulloblastoma, ependymoma, high-grade glioma), sarcomas (osteosarcoma, rhabdomyosarcoma, Ewing sarcoma), leukemias, hepatoblastoma, and Neuroblastoma (NB). Beyond their role in oncogenesis, somatic activating PIK3CA mutations also drive a spectrum of vascular anomalies and overgrowth disorders, providing the biological rationale for targeted PI3K inhibition [89,90,91].

Alpelisib (Novartis; Piqray^R^) is a selective inhibitor of class I PI3K with preferential activity against the p110α catalytic subunit encoded by PIK3CA. Preclinical studies in an inducible PIK3CA-driven mouse model of congenital lipomatous overgrowth, vascular malformations, epidermal nevi, scoliosis/skeletal and spinal syndrome (CLOVES), a phenotype within the PIK3CA-related overgrowth spectrum (PROS), demonstrated that Alpelisib effectively inhibited PI3K/AKT/mTOR signaling. It prevented disease development when administered before induction of mutant PIK3CA and ameliorated established manifestations when initiated after disease onset. These therapeutic effects were dependent on continuous treatment, as drug withdrawal resulted in recurrence of the disease phenotype [92]. 

Efficacy of alpelisib was established in the EPIK-P1 study, a single-arm, non-interventional real-world study evaluating pediatric and adult patients with severe PROS treated with single-agent Alpelisib. At week 24, 27% of the overall cohort—and 37.5% of patients with evaluable imaging—achieved a ≥20% reduction in target lesion volume, with 60% of responders maintaining benefit for at least 12 months [93]. In addition to reducing lesion volume, treatment was associated with clinically meaningful improvements in PROS-related symptoms, quality of life and functional outcomes, while decreasing the need for invasive procedures and treatment related-morbidity compared with previous therapies. Clinical benefit was observed across a broad spectrum of PIK3CA-driven vascular anomalies, including those refractory to previous standard therapies, with an overall favorable safety profile. These findings led to FDA approval of Alpelisib in April 2022 for patients ≥2 years with severe manifestations of PROS requiring systemic therapy, making it the first PI3Kα inhibitor approved for a pediatric indication [94]. This approval expanded in 2024 to include treatment of selected PIK3CA-related vascular malformations such as lymphatic, venous, and mixed anomalies, often refractory to conventional interventions. Subsequent pediatric studies have reinforced the clinical benefit of Alpelisib in these disorders, demonstrating sustained reductions in lesion size and clinically meaningful improvements in functional outcomes, particularly in children with complex head and neck lymphatic malformations [95].

The recommended adult dose of Alpelisib for the treatment of PROS is 250 mg orally once daily. In patients 2 to 17 years old, the recommended starting dose is 50 mg once daily. In pediatric patients ≥ 6 years old who have received Alpelisib for at least 24 weeks, the dose may be increased to 125 mg once daily based on clinical response and may be gradually titrated to the recommended adult dose of 250 mg daily upon reaching adulthood. Serious AEs have been reported in 12% of patients, most commonly dehydration and cellulitis. Importantly, no patients permanently discontinued Alpelisib because of treatment-related AEs. Temporary dose interruptions due to an AE occurred in 11% of patients, whereas dose reductions occurred in 5% of patients primarily because of alopecia, memory impairment, and soft tissue infection [96].

#### PCCB Experience

Our institutional experience includes 18 pediatric patients with refractory vascular tumors treated with alpelisib, of whom 11 have demonstrated substantial clinical benefit including reduction in lesion size, pain control and improved mobility. Two patients who had previously developed severe toxicity with first-line therapies, including mTOR inhibitors, were successfully switched to alpelisib, with significant reduction in treatment-related toxicity.

### 2.5. Selpercatinib for RET-Mutant Thyroid Cancers

Differentiated thyroid carcinoma (DTC) in children and young adults frequently presents as advanced locoregional disease, including a high incidence of cervical lymph node involvement and distant metastases at diagnosis. Despite this aggressive initial presentation, long-term outcomes are excellent, with disease-specific mortality remaining exceptionally low [97,98]. Unlike adult DTC, which is predominantly driven by point mutations such as *BRAF* V600E, pediatric DTC is characterized by a high prevalence of oncogenic kinase fusions, particularly involving *RET* and *NTRK1/3*. The distribution of these molecular alterations is strongly age-dependent, with gene fusions predominating in younger children, whereas *BRAF* and *RAS* mutations become increasingly prevalent during adolescence and young adulthood [99]. Beyond defining distinct molecular subtypes, these molecular alterations also carry prognostic significance. *RET* and *NTRK* fusions, as well as *BRAF* V600E mutations, have generally been associated with more extensive disease at presentation and an increased risk of persistent or recurrent disease, whereas *RAS*-mutant tumors generally follow a more indolent clinical course [100]. Conversely, DICER1-associated thyroid carcinomas generally demonstrate more indolent clinical behavior and are associated with an excellent prognosis [101]. These observations underscore the growing importance of molecular profiling for risk stratification, prognostication, and the selection of targeted therapies in pediatric DTC.

Selpercatinib (Eli Lilly, Retevmo^®^) is a highly selective, orally available small-molecule inhibitor of the RET receptor tyrosine kinase that targets both activating RET point mutations and RET gene fusions. By selectively inhibiting aberrant RET signaling, Selpercatinib suppresses downstream pathways that drive tumor cell proliferation, survival, and progression, while minimizing off-target kinase inhibition [19]. 

Selpercatinib initially received accelerated FDA approval in 2020 for adults with metastatic RET fusion-positive non-small-cell lung cancer and for adult and pediatric patients ≥12 years with advanced or metastatic RET-mutant medullary thyroid carcinoma (MTC) or RET fusion-positive thyroid cancer requiring systemic therapy [102]. In 2022, the FDA granted the first tumor-agnostic approval for Selpercatinib for adults and children aged ≥2 years with locally advanced or metastatic RET fusion-positive solid tumors requiring systemic treatment and lacking satisfactory alternative therapeutic options [19]. Subsequent regulatory updates in 2024 expanded its indication to include pediatric patients aged ≥2 years with advanced or metastatic RET fusion-positive thyroid cancer refractory to radioactive iodine.

The approval of Selpercatinib was primarily supported by the phase I/II, multicenter, open-label LIBRETTO-001 trial, which demonstrated high ORRs and durable responses in patients with RET-altered cancers, including treatment-naïve and previously treated populations [103]. In pediatric and adolescent patients, data from the LIBRETTO-121 study confirmed clinically meaningful antitumor activity, with an ORR of approximately 60%, durable clinical benefit, and a manageable safety profile, supporting the expansion of Selpercatinib to younger age groups [104].

Adverse events associated with Selpercatinib are generally manageable, and treatment discontinuation due to toxicity is uncommon. The most frequently reported treatment-related AEs include QT interval prolongation, elevation in hepatic transaminases, and, less commonly, chylous effusions or chylous ascites, which are thought to result from impaired lymphatic endothelial function secondary to RET inhibition [103,105,106].

#### PCCB Experience

At our institution, one patient with radioiodine-refractory papillary thyroid carcinoma harboring a PTC3–RET fusion was treated with Selpercatinib. Treatment resulted in sustained disease stabilization, partial regression of metastatic lesions, and restoration of radioiodine avidity, enabling subsequent radioactive iodine retreatment, with sustained disease control thereafter. Selpercatinib was well tolerated overall; however, a transient episode of treatment-related nephrotoxicity required dose reduction, with complete renal recovery following dose adjustment. This experience further supports the emerging role of selective RET inhibition as a strategy to induce tumor redifferentiation, restore radioiodine sensitivity, and improve therapeutic outcomes in pediatric DTC.

### 2.6. Crizotinib and Lorlatinib for ALK-Driven Tumors

Anaplastic lymphoma kinase (ALK) is an orphan receptor tyrosine kinase (RTK) originally identified as part of the t(2;5)(p23;q35) chromosomal translocation that generates the NPM1–ALK fusion, a hallmark of most anaplastic large cell lymphomas (ALCL) and a subset of T-cell non-Hodgkin’s lymphomas (NHL) [107]. In many cancers, chromosomal rearrangements involving the ALK locus at chromosome 2p23 generate fusion proteins with a variety of partner genes, resulting in constitutive activation of the ALK kinase domain and downstream signaling pathways that promote tumor cell proliferation and survival [108]. ALK fusions have been identified in various pediatric solid tumors, including inflammatory myofibroblastic tumors (IMT). In contrast, activating germline mutations in ALK represent the major genetic cause of hereditary NB, while somatic ALK mutations occur in approximately 10% of sporadic NB and can also be acquired during NB development and progression [109,110,111,112].

ALCL accounts for approximately 10–15% of pediatric NHL. Approximately 90% of pediatric ALCLs are ALK-positive and harbor chromosomal rearrangements involving the ALK gene, most commonly NPM1::ALK fusion that leads to constitutive activation of ALK [113]. Although conventional multiagent chemotherapy cures approximately 80% of patients, those with R/R disease continue to have a poor prognosis, highlighting the need for novel targeted therapies such as ALK inhibitors [114,115].

IMT is a rare mesenchymal tumor that predominantly affects children, adolescents and young adults. IMTs most frequently arise in the lung, abdomen or pelvis, although virtually any anatomical site may be involved. Complete surgical resection remains the standard treatment whenever feasible [116]. Approximately 50% of IMTs harbor ALK aberrations, providing a strong biological rationale for the use of ALK inhibitors in patients with unresectable, recurrent, or metastatic disease [117].

Crizotinib (Pfizer, Xalkori^®^) is a first-generation TKI that inhibits ATP binding to the kinase domains of ALK, ROS1, and MET. As the first ALK inhibitor to demonstrate clinically meaningful activity in pediatric malignancies, it remains the only ALK inhibitor currently approved for pediatric ALK-positive ALCL and IMT. The phase I/II single-arm ADVL0912 clinical trial (NCT00939770) evaluated Crizotinib in monotherapy and included pediatric patients with relapsed/refractory ALK-positive ALCL or ALK-driven solid tumors [118]. Among the 26 pediatric ALCL patients the ORR was 88%, while the 14-patient IMT cohort showed an ORR of 86%. These results supported the FDA approval of Crizotinib early in 2021 for pediatric patients aged ≥1 year and older and young adults with relapsed or refractory ALK-positive systemic ALCL [119], followed in 2022 by its approval for adults and children aged ≥1 year with unresectable, recurrent, or refractory ALK-positive IMT [120]. The EMA initially approved Crizotinib in 2022 for children aged 6 to <18 years with ALK-positive ALCL and unresectable ALK-positive IMT [121]. In July 2024, this indication was expanded to include children aged ≥1 year. In Spain, the first approval by the AEMPS came in November 2022, extending its use to children aged ≥1 year following the EMA update in 2024. 

Lorlatinib (Pfizer, Lorbrena^®^) is a third-generation macrocyclic TKI targeting ALK and ROS1. It was specifically designed to achieve high CNS penetration and to overcome resistance mutations that limit the activity of first- and second-generation ALK inhibitors, including the F1174 and F1245 variants associated with primary resistance to crizotinib [122]. The first-in-child phase I trial NANT2015-02 (NCT03107988) evaluated Lorlatinib as monotherapy and in combination with topotecan and cyclophosphamide in patients with relapsed or refractory ALK-driven NB [123]. The recommended phase II dose (RP2D) was established at 115 mg/m^2^ once daily for children (<18 years) and 150 mg once daily for adults. Single-agent ORRs were 30% in children and 67% in adults. In combination with chemotherapy, ORR increased to 63% in children, with 48% of responders achieving complete metaiodobenzylguanidine (MIBG) response. The most common AEs included hypertriglyceridemia, hypercholesterolemia, and weight gain. Neurobehavioral AEs occurred mainly in adults and were generally manageable with dose modifications. 

Based on the encouraging results of phase I results, Lorlatinib has been incorporated into the ongoing pivotal COG phase III ANBL1531 trial (NCT03126916) evaluating its addition to frontline therapy for patients with newly diagnosed high-risk (HR) NB harboring ALK alterations. Current NCCN guidelines recommend ALK inhibition for relapsed or refractory ALK-driven HR-NB, whereas the incorporation of Lorlatinib into first-line treatment remains investigational and is currently recommended only within the context of a clinical trial [124]. Although approved for selected adult ALK-positive malignancies, the FDA prescribing information states that the safety and effectiveness of Lorlatinib in pediatric patients have not been established. Accordingly, its use in this setting is currently restricted to clinical trials, expanded access programs, or off-label administration [125].

#### PCCB Experience

We used on- and off-label Crizotinib in three patients: one with IMT, one with malignant peritoneal mesothelioma, and one with ALK-altered HR-NB. Additionally, we have treated 22 patients with ALK-mutated/ALK-amplified HR-NB with off-label Lorlatinib, as well as one ALK-driven infant-type hemispheric glioma who was sequentially treated with Alectinib followed by Lorlatinib, as previously reported [126]. Clinical outcomes are being prospectively collected within the cooperative SACHA study.

### 2.7. Difluoromethylornithine for High-Risk Neuroblastoma

Neuroblastoma is the most common type of cancer in the first year of life and stands as the most frequent extracranial solid tumor of childhood. Approximately 50% of all cases present with HR-NB, which remains associated with poor outcomes despite multimodal intensive treatment regimens, including chemotherapy, maximal safe surgical resection, high-dose chemotherapy with autologous stem cell transplant (ASCT), radiation therapy, and anti-GD2 antibodies [127,128,129]. Current treatment strategies achieve a 5-year EFS rate of 40–60% and OS of 55–75%. However, even among children who complete frontline therapy successfully, the best survival data reported by the COG showed that EFS from the start of immunotherapy declined from 66% at 2 years to 59% at 4 years, demonstrating the need for more effective post-consolidation strategies [130]. Prevention of post-therapy relapses may result in significant improvement of survival for HR-NB patients.

Neuroblastoma, particularly MYCN-amplified disease, is highly dependent on polyamine metabolism. Genes involved in generating polyamines, such as ornithine decarboxylase (OdC), are transcriptionally activated by the MYC family of proteins, thereby maintaining the polyamine levels required for tumor growth in MYC-driven cancers like NB [131,132]. This led to the hypothesis that polyamine metabolism provides a druggable signaling module downstream of MYC. 

Difluoromethylornithine (DFMO; Eflornithine), an irreversible inhibitor of OdC, can antagonize polyamine synthesis, deplete intracellular polyamine pools, and inhibit the growth of MYC-deregulated tumors in preclinical models [133,134,135,136]. Furthermore, *ODC1*, located within 5 Mb of MYCN on chromosome 2p, is co-amplified with *MYCN* in approximately 15–20% of MYCN-amplified NB, corresponding to about 8% of all HR-NB cases [137]. Beyond its antiproliferative effects, ODC inhibition by DFMO reverses an important cancer stem cell (CSC) pathway by downregulating LIN28 and upregulating Let-7, thereby reducing in vitro neurosphere formation in NB cell lines. In addition, depletion of polyamines within tumor cells and the tumor microenvironment may enhance antitumor immunity by restoring immune effector function [138,139]. These preclinical findings provided a strong biological rationale for evaluating DFMO as a maintenance strategy in HR-NB.

DFMO was investigated by the Beat Childhood Cancer (BCC) Consortium in the single-arm, phase II NMTRC003B trial, suggesting improved survival outcomes compared with historical controls from the COG ANBL0032 phase III trial [140]. NMTRC003B enrolled patients with HR-NB who had achieved CR after standard upfront or refractory/relapse treatment and received oral DFMO (750 +/− 250 mg/m^2^ twice a day) for up to 2 years as maintenance treatment. Among patients treated after completion of immunotherapy, the 4-year EFS was significantly higher in the DFMO cohort (84%; *n* = 92) than in the historical no-DFMO control group (72%). These findings were further supported by propensity score-matched analyses designed to minimize baseline differences between patient populations [141]. This study led to the FDA approval of DFMO (USWM, Iwilfin^R^) in December 2023 for adult and pediatric patients with HR-NB who have achieved at least a partial response following completion of multimodal therapy, including anti-GD2 immunotherapy [142].

The most frequently reported AEs with an incidence of ≥5% include sensorineural hearing loss (approximately 13% with new or worsening audiologic impairment), otitis media, pyrexia, pneumonia, and diarrhea. Hematologic toxicities are uncommon and generally mild, with reported rates of grade 3 or 4 neutropenia, anemia, and thrombocytopenia. Laboratory abnormalities were usually related to liver transaminase elevation. Permanent discontinuation of DFMO due to AEs occurred in approximately 11% of patients, frequently associated with ototoxicity or gastrointestinal adverse reactions. Overall, DFMO demonstrates a favorable safety and tolerability profile, supporting its use as a long-term maintenance strategy in HR-NB [143].

#### PCCB Experience

Our institutional experience with DFMO comprises 30 patients with HR-NB treated since May 2024, when the first patient in Spain received DFMO through a compassionate use program at our center. The cohort includes one compassionate-use patient, 18 patients initially enrolled in the BCC clinical trial, and 13 patients treated through a Norgine expanded access program (two patients transitioned from the clinical trial to the expanded access program and were therefore included in both groups). Overall, 13 patients (43%) initiated DFMO in the first CR, whereas 17 (57%) started treatment after the second or subsequent CR, reflecting a heavily pretreated population. Eight patients (27%) had MYCN-amplified tumors, including one patient with concurrent MYCN and ALK amplification. At the time of reporting, 24 patients (80%) remain without evidence of disease and six (20%) are alive with disease, with no deaths recorded. In our experience, DFMO demonstrated an excellent safety profile, with no grade 3–5 treatment-related toxicities. Five patients had pre-existing platinum-related ototoxicity that remained stable during DFMO treatment in all cases. One patient required temporary dose adjustment, and no patient permanently discontinued DFMO because of ototoxicity.

## 3. Radiopharmaceutical Agents

### 3.1. ^177^Lu-DOTATATE (Lutathera^R^) for Neuroendocrine Cancers

Gastro-entero-pancreatic neuroendocrine tumors (GEP-NETs) are rare and heterogeneous tumors arising from neuroendocrine cells of the gastrointestinal tract and pancreas. A defining feature of most GEP-NETs is the overexpression of the somatostatin receptor subtype 2 (SSTR2) on the tumor cell surface. The high density of SSTR2 expression enables both functional diagnostic imaging using 68Ga-DOTATATE PET/CT and receptor-targeted therapies [144,145].

In 2016, the FDA approved DOTA-DPhel-Tyr-octreotate (Dotatate; Advanced Accelerator Applications, Saint-Genis-Pouilly, France), when linked with ^68^Gallium, for PET imaging of NET. In 2018, the FDA approved linking Dotatate with the β-emitter ^177^Lutetium (Advanced Accelerator Applications; Lutathera^R^) for radiotherapy of adults with metastatic NETs. The combination of therapeutic and diagnostic capabilities of a single ligand, such as Dotatate, has been termed theranostics^144^. As of April 23, 2024, ^177^Lu-DOTATATE is FDA-approved for the treatment of pediatric patients aged 12 years and older with SSTR2-positive GEP-NETs [146]. 

^177^Lu-DOTATATE works by selectively binding to SSTR2, which is a group of G protein-coupled receptors involved in mediating the inhibitory effects of somatostatin on hormone secretion and cell proliferation. These receptors participate in key signaling pathways related to cell growth and apoptosis, including MAPK and protein tyrosine phosphatase pathways [144,145,146,147]. Once bound, the radiopharmaceutical is internalized into tumor cells, where it delivers localized radiation therapy through beta particle emission. This targeted radiation induces DNA damage and subsequent tumor cell death, while minimizing damage to the surrounding healthy tissue. This mechanism allows for reduced systemic toxicity and offers a highly specific therapeutic approach [144,145,146,147].

Pediatric approval was based on safety, pharmacokinetic, and dosimetry data from the phase II NETTER-P study (NCT04711135), which included nine pediatric patients: four with GEP-NETs and five with pheochromocytoma or paraganglioma [148]. Clinical efficacy was extrapolated from the pivotal NETTER-1 trial conducted in adults, which demonstrated significant improvements in PFS and ORRs compared with high-dose octreotide [149]. Clinical benefit in pediatric patients was inferred from the robust efficacy demonstrated in adult populations, including prolonged PFS, higher ORRs, and durable disease control observed in phase III trials.

The safety profile of ^177^Lu-DOTATATE is generally favorable and consistent with its targeted mechanism of action. The most commonly reported AEs include nausea, vomiting, fatigue, and transient hematologic toxicities, particularly lymphopenia and thrombocytopenia [147,148]. Renal toxicity may occur due to renal uptake of the radiopharmaceutical, although concomitant amino acid infusions substantially reduce this risk. Rare but clinically significant long-term AEs, including myelodysplastic syndrome and acute leukemia, have been reported in adult series [149]. In the NETTER-P study, no new safety signals were identified in adolescent patients. The most frequently reported treatment-related AE was lymphopenia, reported in 64% of patients, including grade ≥3 lymphopenia in 45%. No clinically meaningful nephrotoxicity was observed. Overall, the safety profile was consistent with that previously reported in adults, supporting the use of ^177^Lu-DOTATATE in pediatric patients with SSTR2-positive GEP-NETs.

Beyond its approved indication in GEP-NETs, ^177^Lu-DOTATATE has also been investigated in R/R HR-NB [150]. Early clinical experience has demonstrated the feasibility and potential clinical activity of this approach in selected patients, leading to the development of prospective clinical studies, including the ongoing phase II LuDO-N trial evaluating ^177^Lu-DOTATATE in children with HR-NB [151].

#### PCCB Experience

**Figure 5:**
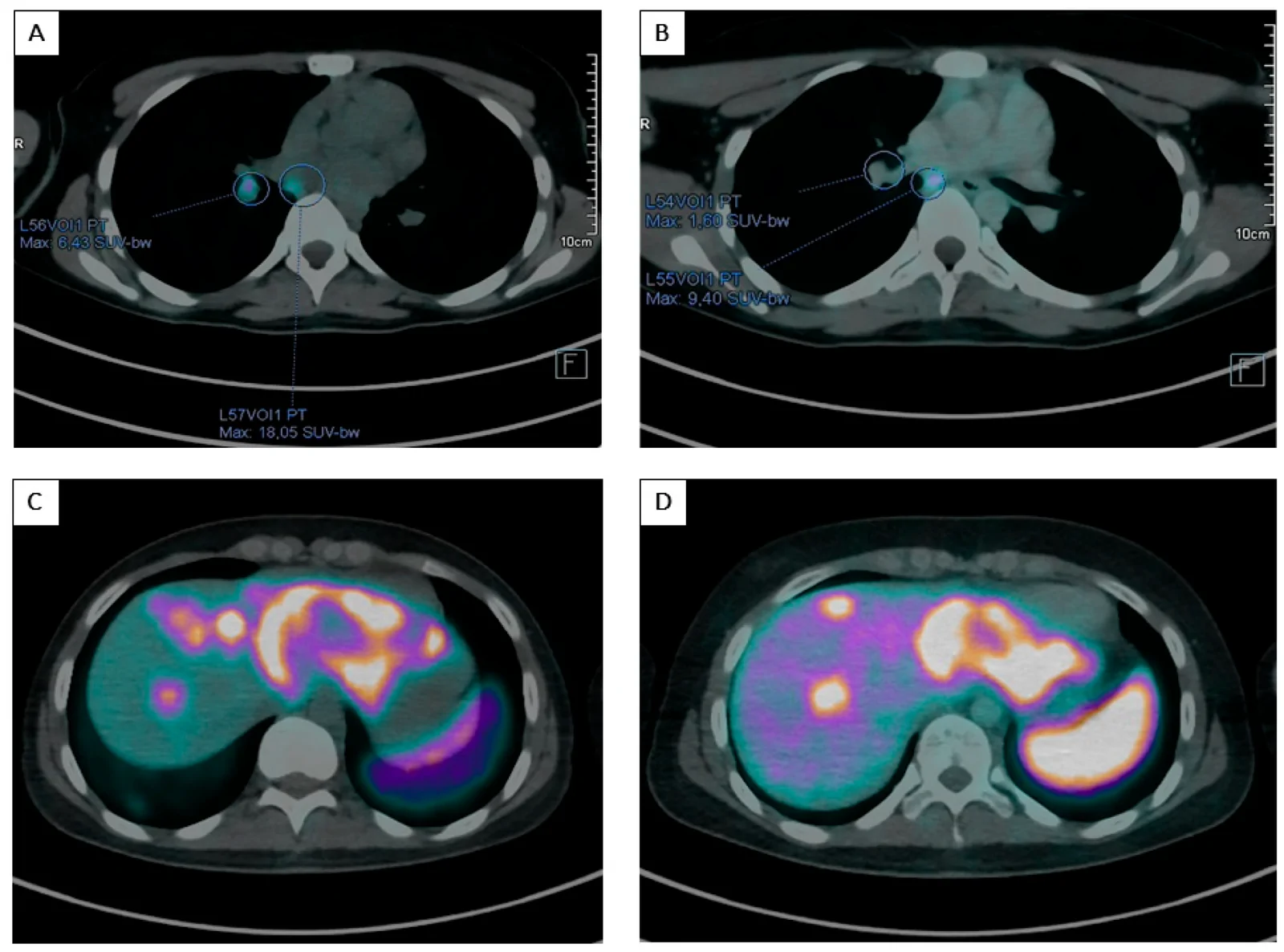
Representative 68Ga-DOTATOC PET/CT images obtained before treatment (**A**,**C**) and after completion of 4 cycles of ^177^Lu-DOTATATE (**B**,**D**) in a 15-year-old patient with metastatic GEP-NET. Follow-up imaging demonstrated decreased somatostatin receptor-avid disease in mediastinal nodal metastases (**A**,**B**) and liver metastases (**C**,**D**), consistent with a partial radiological response and overall disease control.

At the PCCB, we have treated one patient with ^177^Lu-DOTATATE for its approved indication. Our index case is a 15-year-old female diagnosed with stage IV GEP-NET. Radiological reassessment 6 weeks after diagnosis documented progression of hepatic lesions, accompanied by worsening clinical symptoms, leading to the initiation of treatment with ^177^Lu-DOTATATE. The patient received four cycles of the radiopharmaceutical administered at 8-week intervals in combination with monthly long-acting octreotide. After four cycles, the patient achieved a marked clinical and biochemical response. Radiological evaluation demonstrated stable disease at the primary tumor site, with a partial response of liver, pulmonary, and nodal metastases (Figure 5). Treatment was well tolerated, with no severe AEs. The patient is currently receiving maintenance treatment with monthly long-acting octreotide and remains on close follow-up.

Moreover, four patients with HR-NB and progressive or refractory disease despite conventional therapies have received ^177^Lu-DOTATATE, including one patient enrolled in the LuDO-N trial. Although transient disease stabilization was observed in one patient, disease progression ultimately occurred in all cases. Thrombocytopenia was the most relevant treatment-related toxicity observed. 

In addition, ^177^Lu-DOTATATE has been administered to eight patients with refractory CNS tumors demonstrating somatostatin receptor expression, including one choroid plexus carcinoma, one CNS NB, four medulloblastomas, and two ependymomas. Although these applications remain outside the currently approved indications, they highlight the potential broader role of theragnostic approaches in pediatric oncology and support the need for further prospective studies, particularly in CNS tumors.

## 4. Immunotherapeutic Targets

### 4.1. CD19 CAR T-Cell Therapy for CD19+ Acute Lymphoblastic Leukemias

B-cell ALL (B-ALL) is the most common malignancy in the pediatric population, with patients in HICs achieving 5-year EFS rates of up to 85% and OS rates of up to 90% [152,153]. However, approximately 20% of patients experience relapse. If relapse occurs after the completion of primary treatment, most children will enter a second CR, and the chance for cure is about 50%. If relapses occur during therapy, the chance of attaining a second CR is 50–70%, with only 20–30% of patients cured.

CD19 is a cell-surface antigen that is present at high density on most B-ALL cells. Different strategies have been developed to genetically modify autologous T cells with an anti-CD19 antibody fragment coupled with intracellular signaling domains of the T-cell receptor, thereby redirecting cytotoxic T lymphocytes to recognize and eliminate B-ALL cells. These chimeric antigen receptor (CAR)-modified T cells have transformed the treatment of R/R B-ALL, achieving high ORRs and durable remissions in pediatric patients with very high-risk disease [154,155].

Tisagenlecleucel is a second-generation CAR T-cell therapy expressing a CAR composed of a CD3ζ signaling domain, responsible for T-cell activation, and a 4-1BB (CD137) costimulatory domain, which enhances T-cell expansion and persistence. Initial clinical evidence came from a single-center phase 1–2a study conducted at the Children’s Hospital of Philadelphia and the University of Pennsylvania, which included 60 children and young adults with R/R B-ALL and reported a CR rate of 93% [154]. These findings were subsequently confirmed in the pivotal single-cohort, phase 2, multicenter ELIANA trial (NCT02435849), which demonstrated an ORR of 81% among 75 patients after a single infusion of tisagenlecleucel [156]. Responses were durable, with EFS and OS rates of 50% and 76% at 12 months and 44% and 63% at 36 months, respectively [157]. 

These results led to the approval of Tisagenlecleucel (CTL019) by the FDA and EMA in August 2017 and August 2018, respectively, for the treatment of children and young adults up to 25 years of age with R/R B-ALL, including post-transplant relapse or second and subsequent relapses [158,159]. Tisagenlecleucel therapy resulted in substantial improvements in survival, and the main toxicities associated with this novel treatment include cytokine release syndrome (CRS), immune effector cell-associated neurotoxicity syndrome (ICANS), prolonged cytopenias, B-cell aplasia and infections [160,161]. Key limitations of this therapy include disease relapses due to antigen loss and limited CAR T-cell persistence, which have led to the development of dual-targeting and next-generation CAR strategies [162].

#### PCCB Experience

Since 2016, we have treated 142 patients with CAR T-cell therapies, including 94 patients with Tisagenlecleucel and 38 patients with the academic CAR19 product ARI-0001, Varnimcabtagene autoleucel (Gene Vector Barcelona). Varnimcabtagene autoleucel is a fully CD19-targeted CAR T-cell product developed by the CAR T-cell program at Hospital Clínic Barcelona. It was approved by the AEMPS in 2021 for adult patients with relapsed or refractory CD19-positive B-ALL and was subsequently granted Priority Medicine designation by the EMA in December 2021 [163,164,165]. Autologous T cells are expanded and transduced ex vivo using lentiviral particles to express a CAR with anti-CD19 specificity, known as ARI-0001 cells. These ARI-0001 cells express a second-generation CAR, which combines the anti-CD19 single-chain variable fragment (scFv) originating from A3B1, an in-house murine monoclonal antibody, conjugated with the transmembrane domain and co-stimulatory domains. Currently, our group is leading the clinical development of Varnimcabtagene autoleucel in pediatric patients, with the aim of obtaining approval by the EMA.

In addition, we have treated nine patients in clinical trials with other CD19-directed CAR T-cell products or CAR T cells directed against other targets, such as CD1a for cortical T-ALL/lymphoblastic lymphoma.

### 4.2. Blinatumomab for CD19+ Acute Lymphoblastic Leukemias

Blinatumomab is a bispecific T-cell engager (BiTE) antibody construct that targets the CD19 antigen expressed on the surface of B-lineage cells and redirects CD3-positive T cells to induce selective lysis of CD19-positive cells [166]. Blinatumomab was initially developed for the treatment of relapsed or refractory B-ALL, where it demonstrated significant improvements in clinical outcomes in both adults and children [166,167]. The phase 1–2 MT103-205 trial evaluated blinatumomab in pediatric, adolescent, and young adult patients with R/R CD19-positive B-ALL [168]. The study demonstrated high ORRs and clearance of minimal residual disease (MRD) in heavily pretreated patients, allowing many to proceed to allogeneic hematopoietic stem cell transplantation (HSCT). The phase 3 COG AALL1331 trial evaluated the addition of blinatumomab to post-reinduction chemotherapy in children and young adults with low-risk, first-relapse B-ALL [169]. Comparable OS was observed in the overall study population, while blinatumomab significantly improved outcome in patients with bone marrow ± extramedullary relapse. More recently, the phase III COG AALL1731 trial demonstrated that the addition of blinatumomab to chemotherapy significantly improved 3-year OS compared with chemotherapy alone (96% vs. 87.9%, respectively, *p* < 0.001) [170]. These findings supported the integration of blinatumomab into earlier treatment phases for pediatric standard-risk B-ALL. The main clinical benefits include effective MRD eradication, induction of deep remissions in relapsed or refractory disease, reduced exposure to intensive chemotherapy, and its use as a bridge to HSCT or as part of consolidation strategies. 

Blinatumomab has a distinct toxicity profile, mainly related to immune activation. Infusion-related reactions and CRS may occur early after treatment initiation and can be severe, particularly in patients with high tumor burden. Neurological toxicity, including ICANS, is a key complication and may range from headache, tremor or dizziness to encephalopathy, seizures or altered consciousness. Serious infections, as well as neutropenia or febrile neutropenia, are clinically relevant and require close monitoring and prompt treatment. Tumor lysis syndrome is uncommon but potentially life-threatening, especially in patients with high disease burden. Other clinically relevant AEs include transient elevations in liver enzyme and, rarely, severe pancreatitis [166,171].

Blinatumomab (Amgen, Blincyto^®^) was approved by the FDA in September 2016 for pediatric patients with relapsed or refractory Philadelphia chromosome-negative (Ph-negative) B-ALL [172]. On March 2018, Blinatumomab received accelerated approval for adults and pediatric patients with MRD ≥0.1% in first or second CR. Two years later, in August 2020, the EMA approved blinatumomab for pediatric patients (≥1 year) for R/R CD19+ Ph-negative B-ALL after ≥2 prior therapies or prior allogeneic HSCT [173]. In 2024 the FDA approved an expanded indication for its use during consolidation chemotherapy in adult and pediatric patients (≥1 month) with CD19+ Ph-negative B-ALL, regardless of MRD status. On January 2025, the EMA also approved an expanded indication for consolidation in patients with CD19+ Ph-negative B-ALL. This included pediatric patients (≥1 month) with refractory disease or relapse after ≥2 prior treatments or after HSCT, pediatric patients in first high-risk relapse as part of consolidation, and adults with CD19+ Ph-negative B-ALL in consolidation after initial therapy. In Spain, the expanded indications were approved by the AEMPS in May 2025. These include R/R CD19+ B-ALL, eradication of MRD, and use as consolidation therapy after initial treatment, including in pediatrics with high-risk relapse. 

#### PCCB Experience

From 2020 until AEMPS approval in 2025, Blinatumomab was administered to 10 patients including four in R/R disease and six as first-line therapy because of significant toxicity from conventional chemotherapy. Only two of these patients completed two full cycles of blinatumomab. Following AEMPS approval in May 2025, an additional eight patients were treated with blinatumomab by December 2025, including five with R/R disease, one with MRD reappearance, and two treated upfront with blinatumomab because of significant chemotherapy-related toxicity.

Since its approval, the clinical use of blinatumomab has progressively expanded. It has been progressively incorporated into multiple therapeutic scenarios in line with current treatment protocols, with the overall aim of reducing exposure to cytotoxic chemotherapy.

### 4.3. Brentuximab for Hodgkin’s Lymphoma

Hodgkin’s Reed–Sternberg cells are the pathological hallmark of Hodgkin’s Lymphoma (HL), one of the most frequent lymphomas in Western countries. Treatment of HL is one of the greatest success stories in oncology, with protocols including multi-agent chemotherapy and/or radiotherapy achieving cure rates of 80–90% in HICs [174]. 

Hodgkin’s Reed–Sternberg (HRS) cells represent only a small fraction (0.1 to 10%) of the lymph node infiltrates. They are surrounded by a dense inflammatory microenvironment composed of T cells, histiocytes, eosinophils, and plasma cells. These cells recruit immune cells, which in turn provide survival signals that promote HRS cells’ survival and persistence. Therefore, selective targeting of HRS cells represents an attractive therapeutic strategy. CD30 is highly expressed on HRS cells and ALCL cells, whereas its expression in normal tissues is largely restricted to a small population of activated B and T lymphocytes and a subset of eosinophils, making it ideal for selective targeted therapy [174,175].

Brentuximab vedotin is an ADC composed of a monoclonal antibody targeting CD30 linked to the cytotoxic agent monomethyl auristatin E (MMAE). After binding to CD30-expressing cells, the ADC is internalized, releasing MMAE intracellularly and inducing cell-cycle arrest and apoptosis [175]. In the phase 1 study (NCT00430846), Brentuximab vedotin induced durable ORRs in 86% of patients with R/R CD30-positive lymphomas [176]. Treatment was generally well tolerated, with predominantly mild-to-moderate AEs.

Approval of Brentuximab vedotin for pediatric HL was supported by the results of the AHOD1331 trial [177]. This multicenter, randomized phase III study compared Brentuximab vedotin in combination with multi-agent chemotherapy versus standard chemotherapy in patients aged 2–21 years with high-risk classical HL. The trial demonstrated a 3-year EFS of 92% in the Brentuximab vedotin group, compared with 82% in the standard therapy group. Brentuximab vedotin was not associated with significant increases in severe AEs. In addition, the proportion of patients requiring radiotherapy was similar in both treatment groups.

Brentuximab vedotin was approved by both the FDA and the EMA in 2022 for the treatment of pediatric patients aged ≥2 years with high-risk HL, in combination with multi-agent chemotherapy [178,179]. The FDA also approved Brentuximab vedotin in pediatric patients aged 7–18 years with R/R HL or ALCL.

#### PCCB Experience

Nine patients at our center have been treated with Brentuximab vedotin since its approval. Our experience is consistent with the efficacy and favorable safety profile obtained in the clinical trials supporting its approval, particularly in cases of R/R HL.

### 4.4. Naxitamab for High-Risk Neuroblastoma

Neuroblastoma cells carry on their surface high levels of gangliosides and sialic acid containing sugars and proteins, which are important for migration, adhesion and metastasis. GD2 belongs to a unique class of T cell-independent carbohydrate antigens with high density, membrane proximity, homogeneity within and across NB, and rare occurrence of antigen loss [180]. As an oncofetal antigen, GD2 is expressed during fetal development and in mature neurons, pain fibers and skin cells. Two intravenous anti-GD2 IgG monoclonal antibodies (mAbs) had been tested extensively in the clinic, chimeric 14.18 (ch14.18) and mouse 3F8. The mechanism of action of this class molecules involves binding to GD2 and inducing complement-dependent cytotoxicity and antibody-dependent cell-mediated cytotoxicity (ADCC), leading to tumor cell lysis. Most recently, a humanized monoclonal antibody of the IgG1 family was derived from mouse 3F8, naxitamab [181].

Pivotal phase II trials (NCT03363373, NCT01757626) demonstrated that naxitamab in combination with granulocyte–macrophage colony-stimulating factor (GM-CSF) produces clinically meaningful responses in patients with R/R HR-NB. The international, multicenter, phase 2 clinical trial by Mora et al. [182], for patients with R/R HR-NB exclusively affecting the bone and/or bone marrow compartments, showed an ORR of 50% (with CR rate of 38%). Responses were observed in both the bone (ORR 58%, CR 40%) and the bone marrow compartments (ORR 74%, CR 74%). One-year OS and PFS were 93% and 35%, respectively. Similarly, in the study by Kushner et al. [183], patients with refractory osteomedullary disease achieved a CR rate of 75%, responses confirmed by the International NB response criteria and functional imaging studies. The 5-year OS and PFS rates were 64% and 38%, respectively. Overall, these findings indicate that naxitamab plus GM-CSF induces clinically meaningful responses in patients with disease confined to bone and bone marrow, with a safety profile compatible with outpatient administration.

Additional data support the use of naxitamab as consolidation therapy in HR-NB patients achieving first CR. Recently published results of our group show that consolidation with naxitamab plus GM-CSF in HR-NB patients achieving first CR after induction without ASCT consolidation is associated with a 5-year EFS of 57.9% and OS of 78.6%, similar to the best outcome data reported by COG [184]. 

Naxitamab (Ymabs Therapeutics, Danyelza^R^) was granted accelerated approval by the FDA in 2020. It is indicated, in combination with GM-CSF, for the treatment of pediatric patients (≥1 year) and adults with R/R HR-NB involving bone or bone marrow, who have achieved a partial response, minor response, or stable disease following prior therapy [181,185].

#### PCCB Experience

The PCCB has established itself as a world-leading center in the clinical use of naxitamab, particularly in patients with R/R disease. Since 2017, we have treated over 300 new patients in clinical trials, as well as through compassionate use programs or approved label indication. The clinical development of naxitamab includes the use of two regimens: naxitamab with GM-CSF alone, and naxitamab combined with chemotherapeutic agents (chemo-immunotherapy). This experience has been extensively reported and presented, including novel protocols of infusion (Step-Up) that have contributed to making the outpatient administration of naxitamab significantly safer and better accepted by the treating community [184,186,187,188].

## 5. Discussion

According to the FDA, “Novel” drugs are new drugs never approved or marketed before (Drugs@FDA). During the period of 2020–2025, 291 novel drugs were listed in the new approvals by the FDA, 96 (31%) of which were in the field of oncology. Out of these 96 (12–22 per year), 53 (55%) have pediatric indication. In the last 5 years, we have treated 688 unique patients with the new drugs described in this review, a significant number of pediatric oncology patients. 

The first lesson learned from this review is that approvals by regulatory agencies do not equate to patients being able to access new drugs. In our center we were able to access and incorporate the general use of 18 (18.75%) of the 96 new oncological drugs with or without pediatric indication. Many hurdles, all different depending on the region of the world and the regulatory bodies involved, prevail. As the PCCB is in the EU region, the next thing demanding attention from Table 2 is that all oncological drug development was driven from and by U.S.A.-based companies or academic groups. Not only that, but all the new drugs here reviewed had an FDA approval first, with a significant delay (2–24 months) in EMA approval (11/18, 61%) or no approval (7/18, 39%) to date. Furthermore, EMA approval does not mean that the drug becomes broadly and immediately accessible or reimbursed to the many different EU countries (as is in the US with FDA approvals). Significant further delays (many years) may occur subsequently as previously highlighted [189]. Many more hurdles have been identified, mostly regulatory and bureaucratic, representing a significant barrier for EU patients accessing innovative treatments, not to mention the rest of the world where most children live [190]. These significant gaps are especially concerning in the EU, where innovation in the biomedical area is significantly lagging—a warning for all EU-based stakeholders to think about how to change this trend.

Out of the 18 new drugs analyzed, five (28%)—Tovorafenib, DFMO, Naxitamab, ARI CART-19, and blinatumomab—have been developed specifically for pediatric indications (LGG, HR-NB, B-ALL) and by academic groups (BCC, Hospital Clinic Barcelona) or companies (Day One Pharmaceuticals and Ymabs Therapeutics) centered in the pediatric population. This is a relevant change from prior eras and shows the increasing interest from the public and private sectors in rare diseases. Academic groups like BCC or Hospital Clinic-SJD and companies such as One Day Pharmaceuticals or Ymabs Therapeutics have been successful in approving drugs for pediatric-only indications. Also notable is that the development of CART-19 or blinatumomab, focused on pediatric indications, has opened new therapeutic opportunities for adult patients as well. These efforts are commendable and should guide initiatives that want to follow successful drug development in rare diseases like pediatric cancers.

The introduction of novel targeted therapies in pediatric oncology has marked a significant shift from conventional cytotoxic approaches toward precision-based treatment strategies. The agents discussed in this review illustrate how selective targeting of oncogenic pathways can translate into meaningful clinical benefit, even in challenging clinical scenarios. However, not all inhibitors of the same pathway behave similarly, and precise molecular characterization is required for each disease to understand which of the targeted agents work better. For instance, we have summarized in this review six MAPK inhibitors (dabrafenib, trametinib, selumetinib, mirdametinib, tovorafenib and cobimetinib) and shown how Cobimetinib works better for histiocytic diseases, whereas Selumetinib and Mirdametinib are better suited for NF1-deletion tumors.

A significant number of patients have benefitted from newly available drugs in our center. Most importantly, diseases with poor outcomes with conventional therapies like HR-NB or relapsed B-ALL have experienced significant improvements in survival. It is remarkable how immunotherapeutic strategies integrated into current chemotherapy backbones have significantly improved outcomes for patients with HR-NB, B-ALL or lymphomas. These experiences should guide the path for improving poor-prognostic pediatric cancers like sarcomas, brain tumors (DMG, HGG, ependymoma and embryonal subtypes), and other rare and highly malignant cancers like rhabdoid tumors, commonly defined by a lack of immunotherapeutic strategies.

Targeted therapies have shown significant benefits, mainly for single-pathway-driven diseases like LCH, vascular tumors, LGG, IMTs and NETs. These diseases are characterized by specific mutations affecting one tumorigenic pathway and lacking genomic instability, which permits prolonged treatments and rechallenges in the chronic course of the disease. In these cases, pediatric patients have truly benefitted from developments generated by adult-cancer-driven innovation. One of the most relevant contributions of these therapies is their ability to achieve sustained disease control with improved tolerability compared with traditional cytotoxic treatments. This is particularly important in pediatric populations, where long-term toxicity and quality of life are key considerations. In addition, targeted therapies may, in selected cases, modify tumor biology, as illustrated by the restoration of sensitivity to radioactive iodine in RET-driven thyroid carcinoma or induced differentiation in HR-NB. Metabolic pathways are being explored, and the first examples of drugs targeting key metabolic nodes like DFMO suggest that there could be more therapeutic opportunities coming up in the future. The same goes for radionucleotide-based therapies, where radiation is delivered more selectively targeted to tumor cells. ^177^Lu-DOTATATE is just the first example of this expanding area of therapeutic opportunities. Combining radioactive cargo with immune targeting strategies (radio immunotherapy) might provide better alternatives to deliver radioactivity more selectively.

Long courses of treatment and new guidelines on how to manage new toxicities are changing the general landscape of how to treat pediatric tumors. New generations of pediatric oncology fellows are learning non-chemotherapy-based strategies for many tumors that are currently treated exclusively with targeted therapy. Recent developments in the management of low-risk ALL suggest that non-chemotherapy-based strategies might soon become standard even in highly malignant tumors like ALL. The future is becoming rapidly brighter compared to stagnant decades prior.

Despite these advances, several challenges remain. First, the development of acquired resistance represents a major limitation in malignant tumors like HGG, often leading to disease progression after an initial response [126]. Second, the optimal integration of new therapies into clinical practice remains an area of active investigation. And third, questions regarding treatment sequence, combination strategies, and long-term safety require further study. 

## 6. Conclusions

In summary, real-world experience, such as the one from the PCCB, complements clinical trial data and offers valuable insights into treatment feasibility and outcomes in routine clinical practice. Recently approved targeted therapies are redefining the management of pediatric cancer by enabling more precise and less toxic treatment strategies. Continued advances in molecular biology and therapeutic development focused specifically on developmental cancers will determine the pace at which we will further improve outcomes. The revolution of pediatric oncology precision medicine has already started and there are no reasons to think that the era of only cytotoxic (chemo and radio) therapy might come to an end.

## Figures and Tables

**Table 1:** Number of approvals.

YEAR	FDA Novel Drug Approval	FDA Oncology Novel Drug	Pediatric Oncology Approvals
2025	46	15	15
2024	50	17	10
2023	55	14	10
2022	37	12	5
2021	50	16	6
2020	53	22	7
Total	291	96 (33%)	53 (55%)

Novel drug therapy approval: “Novel” drugs are new drugs never before approved or marketed in the U.S. See Drugs@FDA for information about all CDER’s approved drugs and biological products. Pediatric Oncology Drug Approvals: These approvals reflect a variety of strategies applied to pediatric drug development. Examples include initial approvals of drugs developed specifically for treatment of pediatric cancers, supplemental approvals of drugs initially developed for adult cancers, approvals based on programs employing extrapolation of adult efficacy data to pediatric populations, and approvals of tumor-agnostic indications.

**Table 2:** New Drugs Reviewed.

Generic Name (Trade Name)		Target/Mechanism of Action	Indications	Age	Date of Approval	Clinical Trial Leading to Indication	Main Results	Ongoing Clinical Trials in Children
**Nirogacestat** Ogsiveo^®^		Selective γ-secretase inhibitor (GSI) that blocks the Notch pathway by preventing Notch receptor activation	Adult patients with progressing desmoid tumors who require systemic therapy	≥18 y	November 2023 (FDA) August 2025 (EMA)	DeFi trial (NCT03785964)	ORR: 41% vs. 8% with nirogacestat vs. placebo PFS: significant benefit over placebo (HR 0.29 (95% CI 0.15–0.55)) EFS: 76% vs. 44% with nirogacestat vs. placebo	NCT04195399
**MAPK** **inhibitors**	**Dabrafenib** Tafinlar^®^ **Trametinib** Mekinist^®^	Dual MAPK pathway blockade by inhibiting mutant BRAF and downstream MEK	LGG with a BRAF V600E mutation (FDA, EMA) HGG with BRAF V600E mutation who have received prior radiation and/or chemotherapy (EMA)	≥1 y	March 2023 (FDA) June 2023 (EMA)	Study CDRB436G2201 (NCT02684058)	ORR: 47% vs. 11% with dabrafenib + trametinib vs. chemotherapy PFS: 20.1 months vs. 7.4 months, respectively	NCT03919071 NCT04201457 NCT06712875
	**Selumetinib** Koselugo^®^	Non-ATP-competitive selective inhibitors of MEK1/2	Symptomatic, inoperable NF1-associated plexiform neurofibromas	≥1 y	April 2020 (FDA) June 2021 (EMA)	SPRINT phase 2 trial (NCT01362803)	ORR: 68% 3-year PFS: 84% vs. 15% in the natural history cohort Median PFS: not reached vs. 1.3 years in controls Clinically meaningful improvements in pain, quality of life, strength, and range of motion	NCT06175637 NCT06188741
	**Mirdameinib** Gomekli^®^			≥2 y	February 2025 (FDA) No formal approval by the EMA	ReNeu trial (NCT03962543)	ORR in children (a ≥20% reduction): 52% Children maximum reduction from baseline of >50%: 52%	NCT07521657 NCT03962543 NCT05028166
	**Tovorafenib** Ojemda™	Selective pan-RAF kinase inhibitor	R/R pediatric LGG harboring: BRAF fusion or rearrangement BRAF V600 mutation with disease progression after prior systemic therapy	≥6 m	April 2024 No formal pediatric approval by the EMA	FIREFLY-1 (NCT04775485)	ORR: 67% DOR: 16.6 months TTR: 3 months	NCT04775485 NCT05566795 NCT06381570 NCT07206849
	**Cobimetinib** Cotellic^®^	MEK1/2 inhibitor	Unresectable or metastatic melanoma with a BRAF V600 mutation (combined with vemurafenib) (FDA, EMA) Histiocytic neoplasms (single-agent) (FDA)	≥18 y	November 2015 (FDA, EMA): melanoma October 2022 (FDA): histiocytic neoplasms	NCT02649972	ORR: 83.3%	NCT04079179
**Belzutifan** Welireg ^®^		Selective HIF-2α inhibitor; key mediator of VHL-associated tumorigenesis	PNET, ccRCC, CNS hemangioblastomas	≥12 y	May 2025 (FDA) No formal pediatric approval by the EMA	LITESPARK-004 (NCT03401788)	ORR: 83% in pNETs, with lower response rates in CNS hemangioblastomas and ccRCC	NCT04195750 NCT05239728
**Alpelisib** Piqray ^®^		Selective inhibitor of the PI3K p110α catalytic subunit, suppressing aberrant PI3K/AKT/mTOR signaling	PROS (FDA, EMA) PRVM (FDA only)	≥2 y	PROS: April 2022 (FDA) September 2023 (EMA) PRVM: August 2024 (FDA)	EPIK-P1 (NCT04285723)	ORR (≥20% reduction in target lesion volume): 37.5%	NCT04085653 NCT06997588
**Selpercatinib** Retevmo^®^		Selective TKI targeting oncogenic RET alterations	RET-mutant MTC RET fusion-positive tyroid cancer refractory to radioactive iodine	≥2 y (FDA) ≥12 y (EMA)	May 2020 (FDA; expanded to ≥2 years in May 2024) February 2021 (EMA)	LIBRETTO-001 (NCT03157128)	ORR: 60%	NCT04320888 NCT03899792
**Crizotinib** Kalkori^®^		ATP-competitive TKI targeting ALK, ROS1, and MET	Relapsed/refractory ALK-positive systemic ALCL Unresectable, recurrent, or refractory ALK-positive IMT	≥1 y (FDA) 1–<18 y (EMA)	ALCL: January 2021 (FDA) August 2022 (EMA) IMT: July 2022 (FDA) August 2022 (EMA)	ADVL0912 (NCT00939770)	ORR: 88% in ALCL and 86% in IMT	CRISP ITCC-053 NCT05160922
**Lorlatinib** *Lorbrena^®^*		Macrocyclic TKI targeting ALK and ROS1 with high CNS penetration	R/R ALK-driven neuroblastoma	No formal pediatric approval		NANT2015-02 (NCT03107988)	ORR: 30% with monotherapy and 63% in combination with chemotherapy Complete MIBG response: 48% in combination with chemotherapy	ANBL1531 (NCT03126916)
**DFMO, eflornithine** Iwilfin^TM^		Irreversible inhibitor of ornithine decarboxylase (ODC), the rate-limiting enzyme in polyamine biosynthesis	High-risk neuroblastoma after multimodal therapy	≥1 y	December 2023 (FDA) No formal pediatric approval by the EMA	Comparative analysis of Study 3b (NCT02395666) and ANBL0032	EFS: 84% vs. 72% with DFMO vs. no-DFMO OS: 96% vs. 84%, respectively	NCT02679144 NCT04301843 ANBL1821
**^177^Lu-DOTATATE** Lutathera ^®^		Radiolabeled somatostatin analog delivering β-radiation following binding to SSTR2	SSTR2+ GEP-NETs	≥12 y	April 2024 (FDA) No formal pediatric approval by the EMA	NETTER-P (NCT04711135) NETTER-1 (NCT01578239)	ORR: 18% vs. 3% with Lutathera + octreotide vs. octreotide alone EFS: 65% vs. 11%, respectively OS: clinical OS benefit (11 months longer). No statistical significance	NCT04711135 NCT06607692
**CAR-T** Tisagenlecleucel, Kymriah^®^		Autologous T cells expressing an anti-CD19 chimeric antigen receptor redirecting cytotoxic T-cell activity against CD19-positive B cells	B-ALL with: Refractory disease Relapse after allo-HSCT Second or later relapse	≤25 y	August 2017 (FDA) August 2018 (EMA)	ELIANA (NCT02435849)	EFS: 44% OS: 63%	NCT03117751 NCT04094311
**Blinatumomab** Blincyto^®^		Bispecific antibody targeting CD19 on B-lineage cells and CD3 on T cells, redirecting T-cell cytotoxicity against CD19-positive cells	CD19-positive B-ALL: Consolidation after multiphase chemotherapy (FDA) First or second complete remission with MRD ≥0.1% (FDA) R/R disease (FDA, EMA *)	≥1 m	September 2016 (FDA) August 2018 (EMA)	MT103-205 (NCT01471782) COG-AALL1331 (NCT02101853) COG AALL1731 (NCT03914625)	CR: 33% in heavily pretreated patients DFS/OS: 73% vs. 54% with blinatumomab vs. chemotherapy in first BM ± extramedullary relapse DFS: 96% vs. 88% with blinatumomab vs. chemotherapy as first-line consolidation in standard-risk B-ALL	NCT04521231 NCT07134088 NCT06124157
**Brentuximab** Adcetris^®^		Anti-CD30 antibody–drug conjugate delivering MMAE to CD30-expressing cells, resulting in microtubule disruption and apoptosis	Previously untreated systemic ALCL Previously untreated high-risk cHL ** R/R cHL	≥2 y	ALCL: November 2021 (FDA) January 2022 (EMA) cHL: May 2022 (FDA) June 2022 (EMA)	COG ANHL12P1 (NCT01979536) COG AHOD1331 (NCT02166463)	Systemic ALCL_ EFS: 79% OS: 97% High risk cHL: EFS: 92% vs. 82% with brentuximab vs. chemotherapy alone.	NCT02166463 NCT01920932 NCT03755804
**Naxitamab** Danyelza^®^		Anti-GD2 monoclonal antibody binding GD2 on tumor cells and inducing ADCC	R/R high-risk neuroblastoma in bone or BM with partial response, minor response, or stable disease after prior therapy (combined with GM-CSF)	≥1 y	November 2020 (FDA) No formal pediatric approval by the EMA	Study 201 (NCT03363373)	ORR: 50% (CRR 38%) PFS: 35% OS: 93%	NCT03363373 NCT05489887 NCT07027748 NCT06540963
						NCT01757626	CRR: 75% PFS: 38% OS: 64%	

ADCC: antibody-dependent cell-mediated cytotoxicity; allo-HSCT: allogenic hematopoietic stem cell transplant; ALCL: anaplastic large cell lymphoma; B-ALL: B-cell acute lymphoblastic leukemia; BM: bone marrow; cHL: classical Hodgkin Lymphoma; CR: complete remission; CRR: complete response rate; DFS: disease-free survival; DOR: duration of response; EFS: event-free survival; GEP-NETs: gastroenteropancreatic neuroendocrine tumors; GM-CSF: granulocyte–macrophage colony-stimulating factor; HGG: high-grade glioma; IMT: inflammatory myofibroblastic tumor; LGG: low-grade glioma; MMAE: monomethyl auristatin E; MRD: minimal residual disease; MTC: medullary thyroid cancer; OS: overall survival; ORR: Overall Response Rate; PFS: progression-free survival; PI3K: Phosphatidylinositol 3-Kinase Alpha; PROS: PIK3CA-related overgrowth spectrum; PRVM: PIK3CA-Related Vascular Malformations; R/R: relapsed or refractory; SSTR2: somatostatin receptor subtype 2; TTR: time to response. * EMA pediatric indications for blinatumomab: Philadelphia chromosome-negative, CD19-positive B-ALL refractory or relapsed after ≥2 prior therapies, or after prior allo-HSCT. High-risk first-relapsed Philadelphia chromosome-negative, CD19-positive B-ALL as consolidation therapy. ** EMA pediatric frontline indication for brentuximab in cHL: Previously untreated stage II CD30-positive cHL with risk factors, or stage III–IV CD30-positive cHL.

## Data Availability

No new data were created or analyzed in this study. Data sharing is not applicable to this article.

## Abbreviations

**Abbreviation**	**Full Term**
PCCB	Pediatric Cancer Center Barcelona
HIC	High Income Countries
Ph+ ALL	Philadelphia chromosome positive Acute Lymphoblastic Leukemia
CML	Chronic Myelogenous Leukemia
APML	Acute Promyelocytic Leukemia
ATRA	All-Trans Retinoic Acid
TRK	Tyrosine Receptor Kinase
HR-NB	High-Risk Neuroblastoma
TCGA	The Cancer Genome Atlas
NIH	National Institute of Health
USA	United States of America
FDA	US Food and Drug Administration
ICI	Immune Checkpoint Inhibitors
PD-1	Programmed cell Death protein-1
dMMR	Mismatch Repair-deficient
NTRK	Neurotrophic Tyrosine Receptor Kinase
RET	Rearranged during Transfection
ADC	Antibody–Drug Conjugate
DTs	Desmoid Tumors
FAP	Familial Adenomatous Polyposis
APC	Adenomatous Polyposis Coli
PFS	Progression-Free Survival
ORR	Objective Response Rate
CR	Complete Response
AEs	Adverse Events
TKI	Tyrosine Kinase Inhibitors
COG	Children’s Oncology Group
LGG	Low-Grade Gliomas
WHO	World Health Organization
MAPK	Mitogen Activated Protein Kinase
FGFR1	Fibroblast Growth Factor Receptor 1
DCR	Disease Control Rate
MRI	Magnetic Resonance Imaging
RANO	Response Assessment in Neuro-Oncology
LCH	Langerhans cell histiocytosis
ECD	Erdheim–Chester disease
PET	Positron Emission Tomography
CNS	Central Nervous System
NGS	Next-Generation Sequencing
NF1	Neurofibromatosis type 1
PN	Plexiform Neurofibromas
CK	Creatine Kinase
VHL	von Hippel–Lindau disease
